# Supplementary material for: Benzenesulfonamides Incorporating Hydantoin Moieties Effectively Inhibit Eukaryoticand Human Carbonic Anhydrases
Source: Int J Mol Sci. 2022 Nov 15;23(22):14115. doi: 10.3390/ijms232214115 (PMC9696710; doi:10.3390/ijms232214115)
Supplement: Supplementary file 1 [file ijms-23-14115-s001.zip › ijms-2008068-supplementary.pdf]

Benzenesulfonamides incorporating hydantoin moieties effectively inhibit eukaryotic and human carbonic anhydrases

Morteza Abdoli[1], Viviana De Luca[2], Clemente Capasso[2], Claudiu T. Supuran*[,3] and Raivis Žalubovskis*[,1,4]

[1] Institute of Technology of Organic Chemistry, Faculty of Materials Science and Applied Chemistry, Riga Technical University, P. Valdenaiela 3, LV-1048 Riga, Latvia
[2] Department of Biology, Agriculture and Food Sciences, Institute of Biosciences and Bioresources, Via Pietro Castellino 111, 80131 Napoli, Italy
[3] NEUROFARBA Department, Pharmaceutical and Nutraceutical Section, University of Florence, Via Ugo Schiff 6, 50019 Florence, Italy
[4] Latvian Institute of Organic Synthesis, Aizkraukles 21, LV-1006, Riga, Latvia
* Correspondence: claudiu.supuran@unifi.it (C.T.S.); raivis@osi.lv (R.Ž.)
* CONTACT Raivis Žalubovskis raivis@osi.lv Latvian Institute of Organic Synthesis, 21 Aizkraukles Str, Riga, LV-1006, Latvia; Claudiu T. Supuran claudiu.supuran@unifi.it Dipartimento Neurofarba, Sezione di Scienze Farmaceutiche e Nutraceutiche, Universitàdegli Studi di Firenze, Sesto Fiorentino, Florence, Italy

SUPPORTING INFORMATION

6

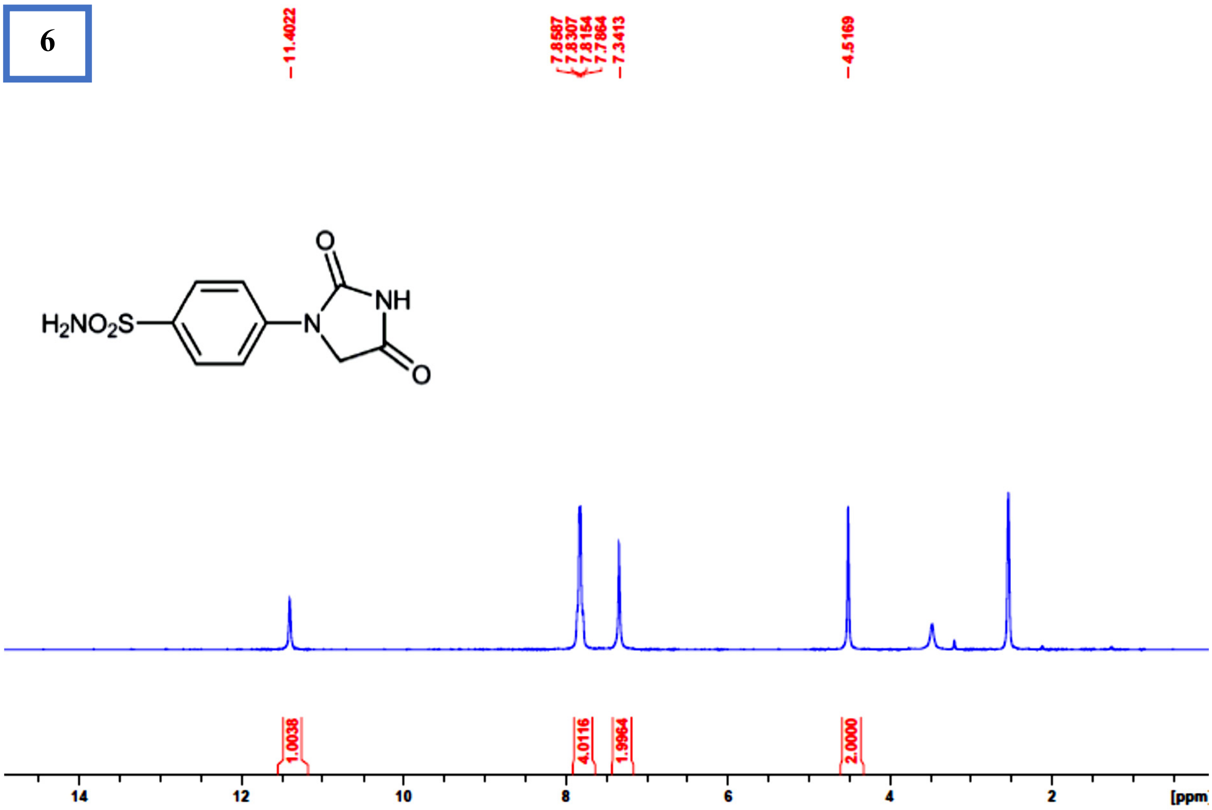
11.4022
7.8587
7.8307
7.8154
7.7864
7.3413
4.5169
H_2NO_2S
N
NH
O
O
1.0038
4.0116
1.9964
2.0000
14
12
10
8
6
4
2
[ppm]

6

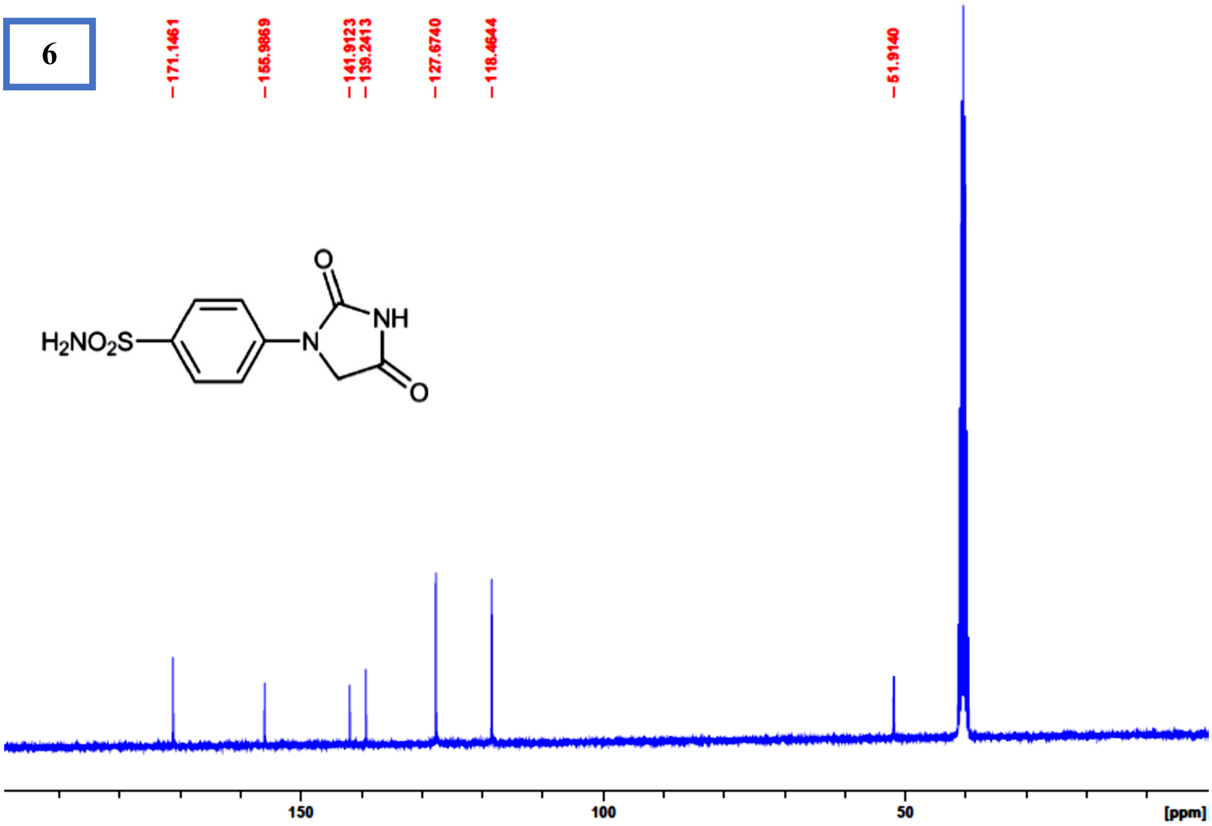
171.1461
155.9869
141.9123
139.2413
127.6740
118.4644
51.9140
H_2NO_2S
N
NH
O
O
150
100
50
[ppm]

8a

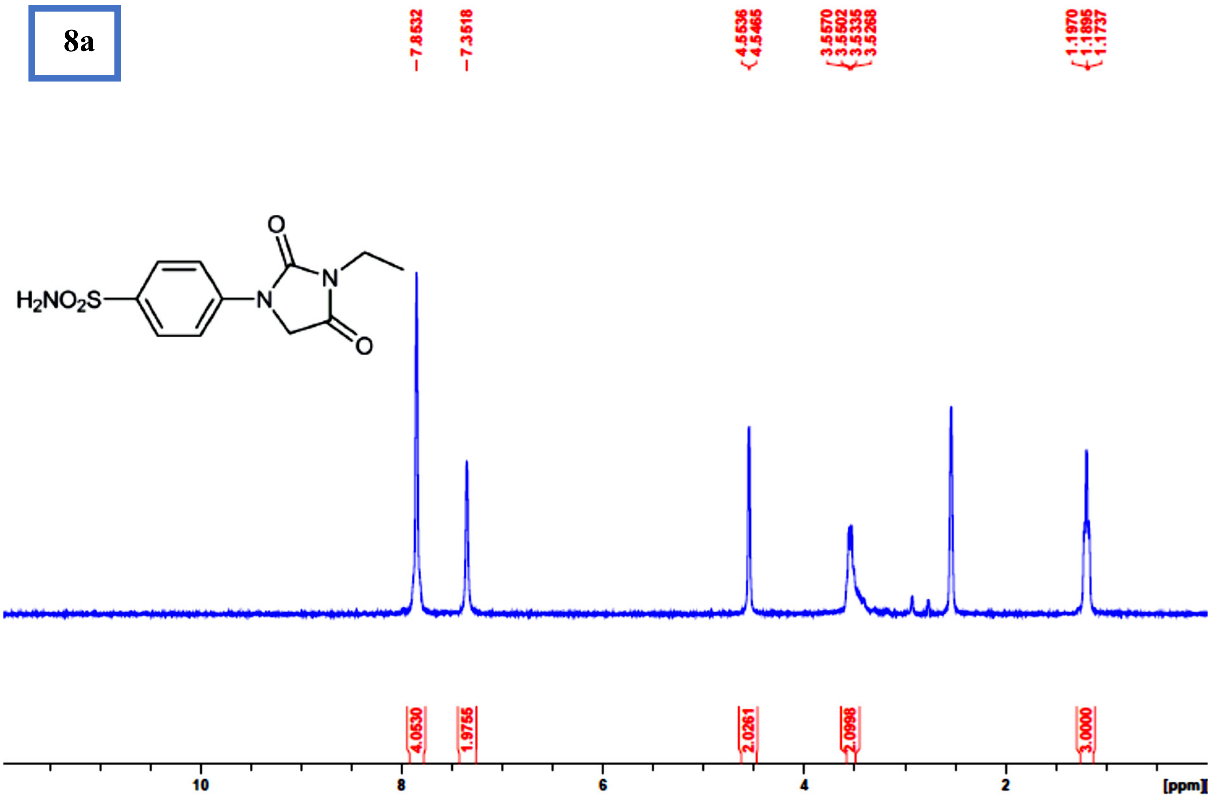

DMSO+D2O

8a

DMSO+D_2O

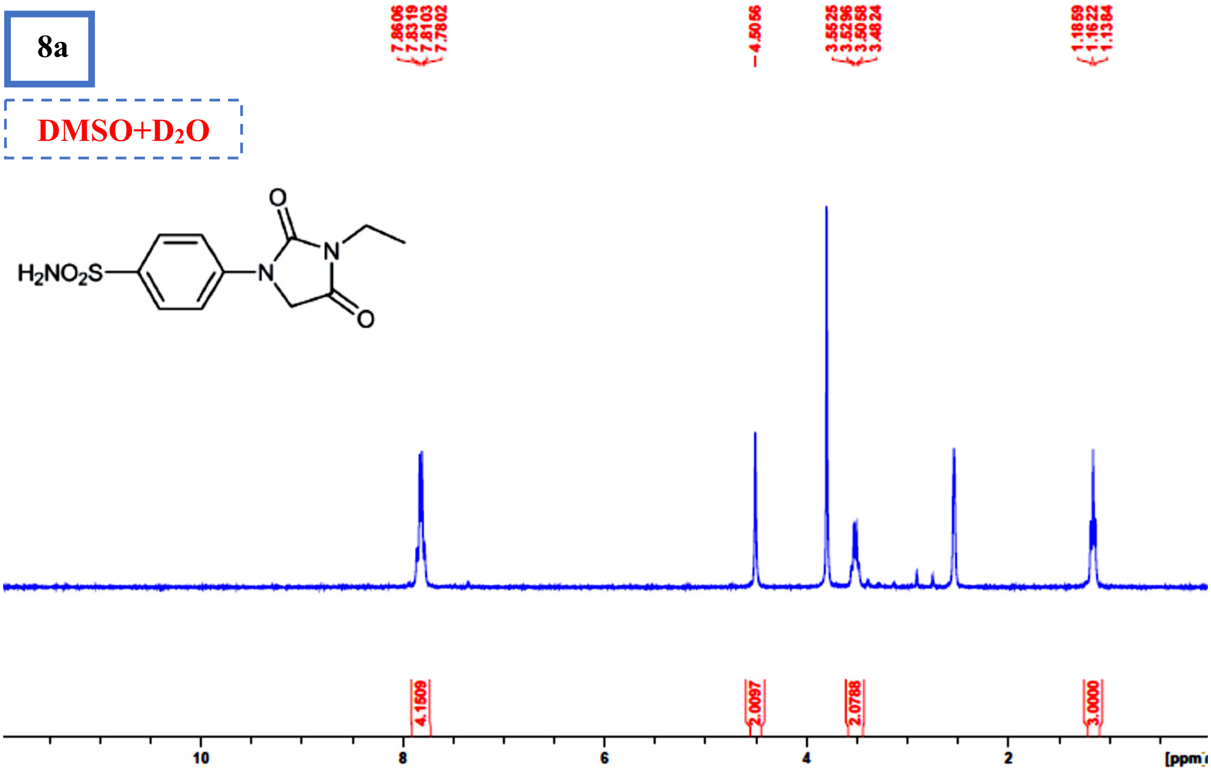

8a

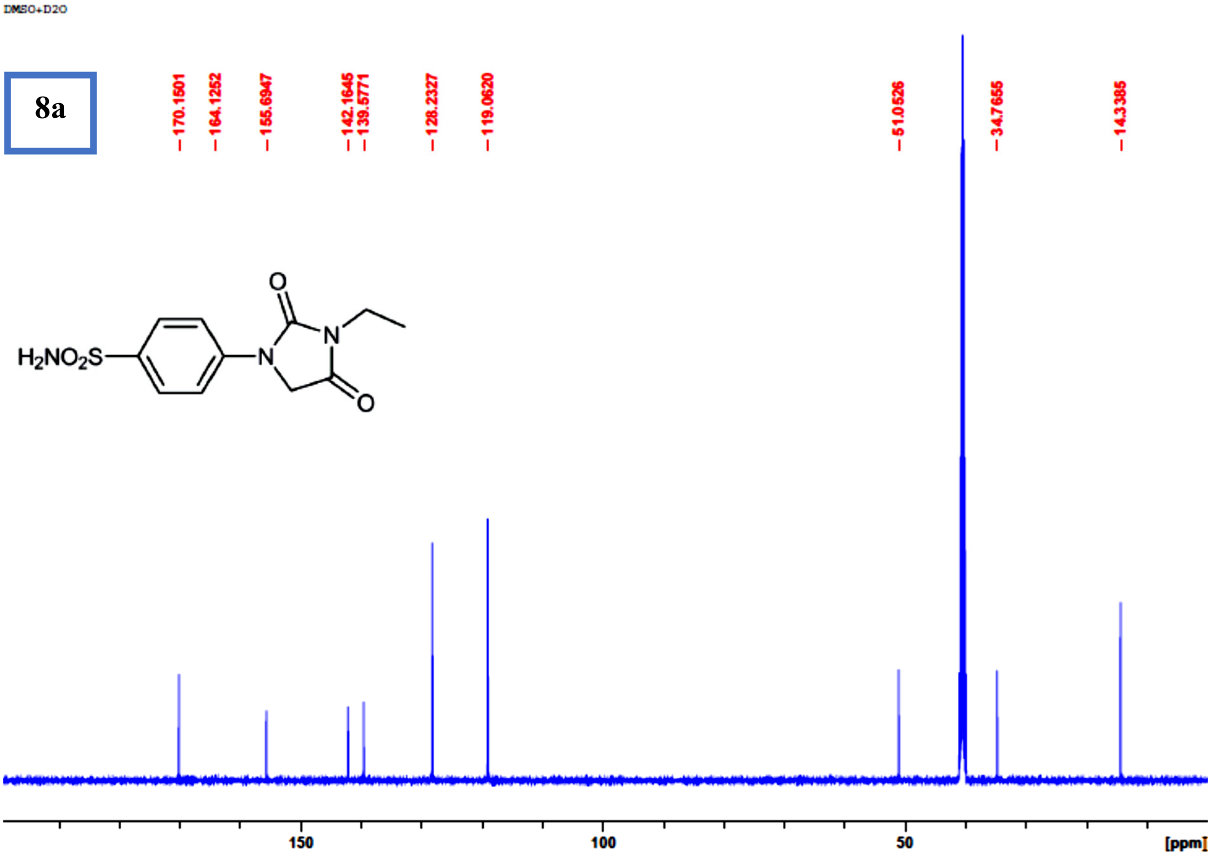
DMSO+D2O
170.1501
164.1252
155.6947
142.1645
139.5771
128.2327
119.0620
51.0526
34.7655
14.3385
150
100
50
[ppm]

8b

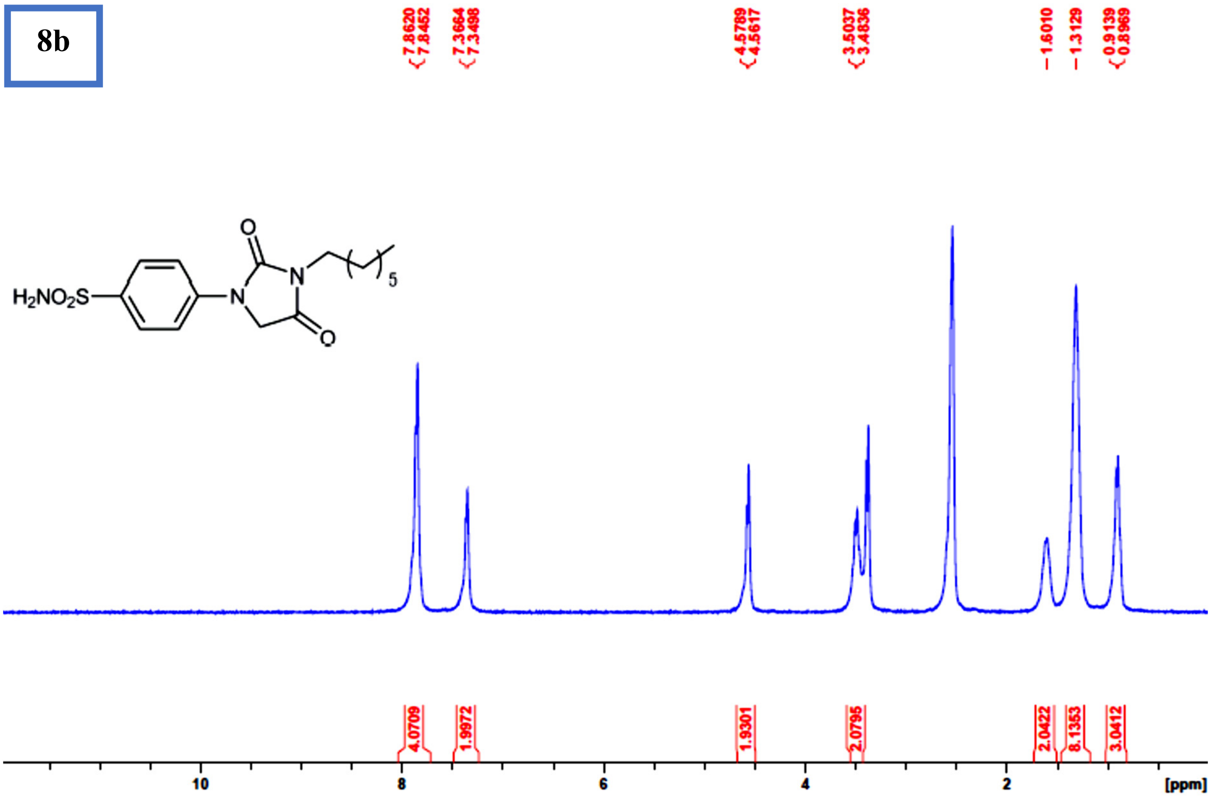
7.8620
7.8452
7.3664
7.3498
4.5789
4.5617
3.5037
3.4836
1.6010
1.3129
0.9139
0.8969
4.0709
1.9972
1.9301
2.0795
2.0422
8.1353
3.0412
10
8
6
4
2
[ppm]

DMSO+D2O

8b

DMSO+D_2O

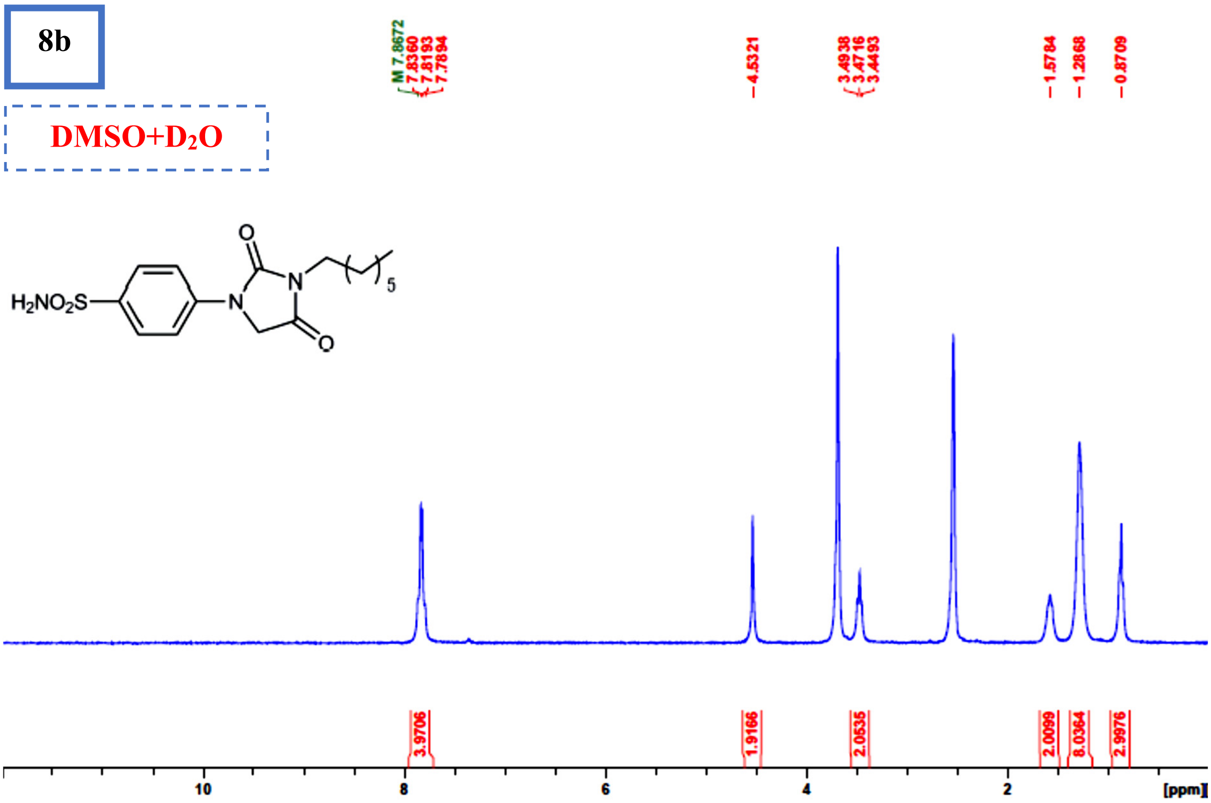

8b

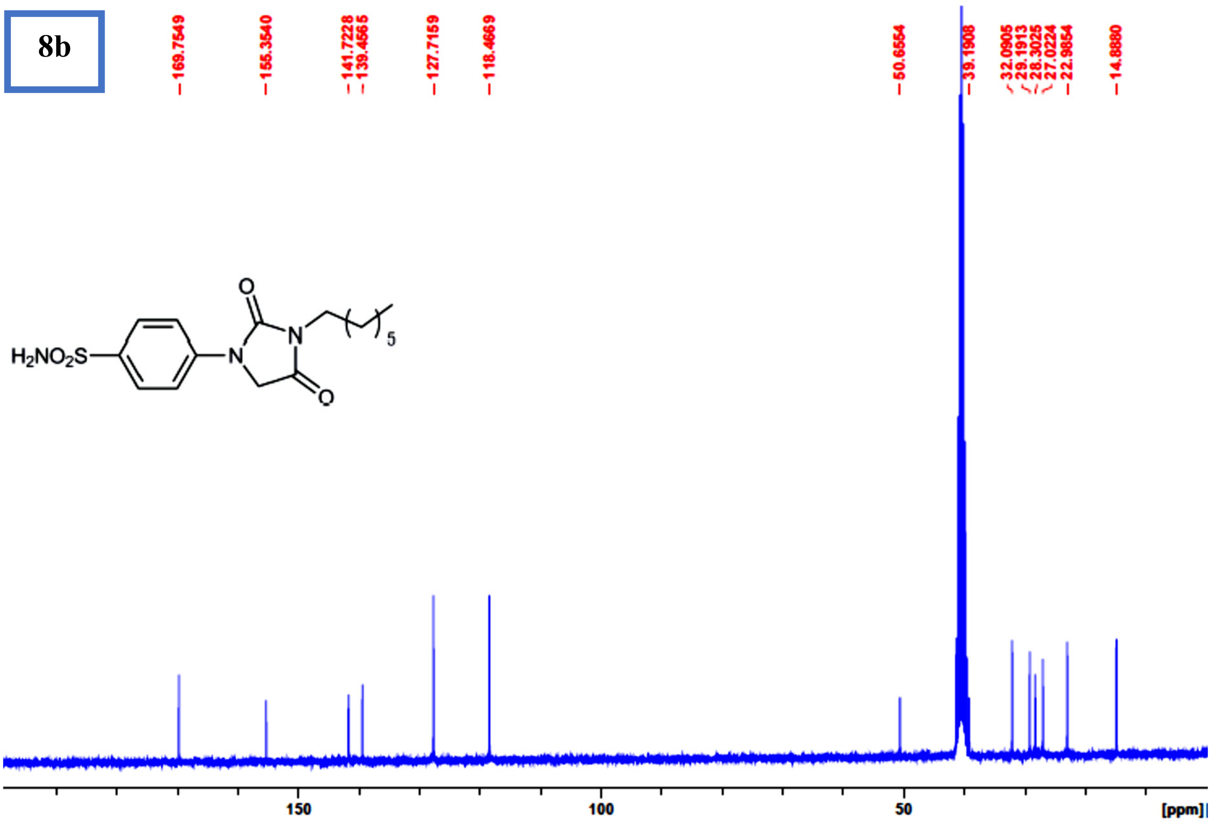

8c

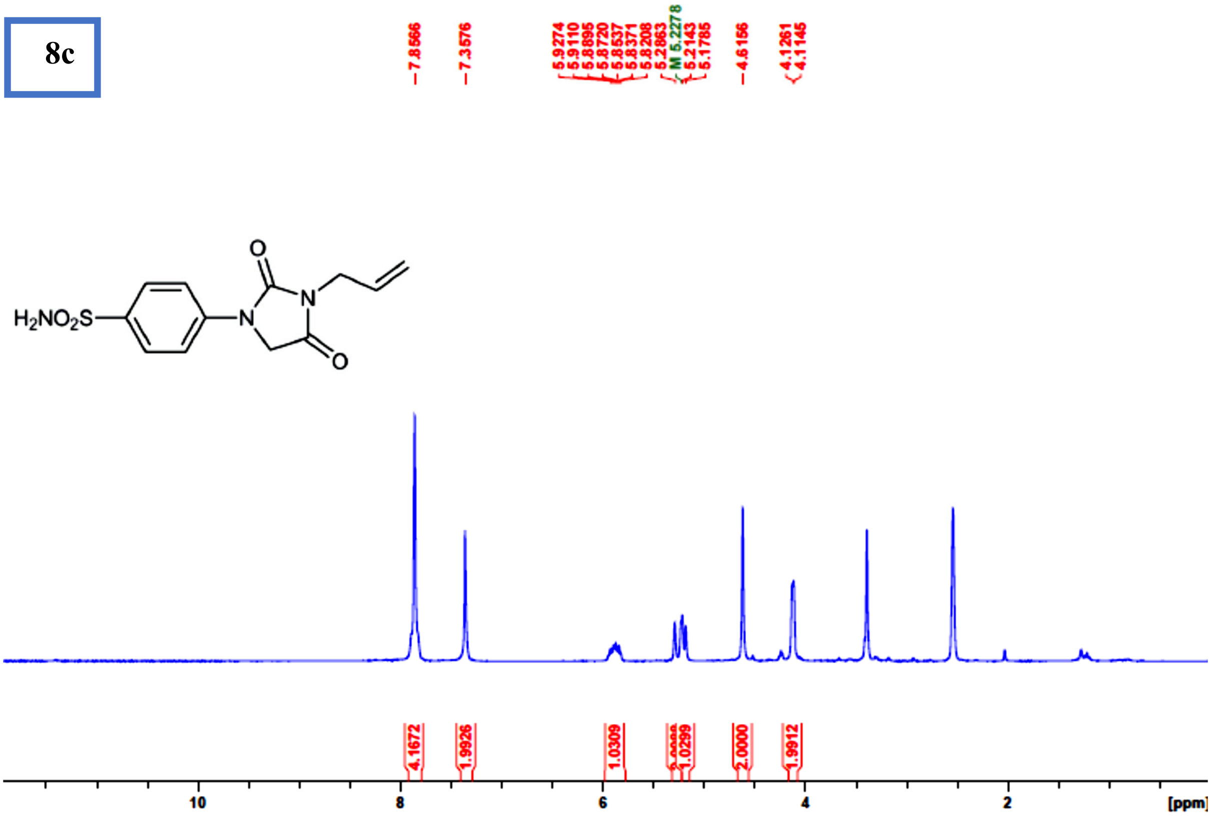
7.8566
7.3576
5.9274
5.9110
5.8895
5.8720
5.8537
5.8371
5.8208
5.2863
M 5.2278
5.2143
5.1785
4.6156
4.1261
4.1145
H2NO2S
N
O
4.1672
1.9926
1.0309
1.0299
2.0000
1.9912
10
8
6
4
2
[ppm]

8c

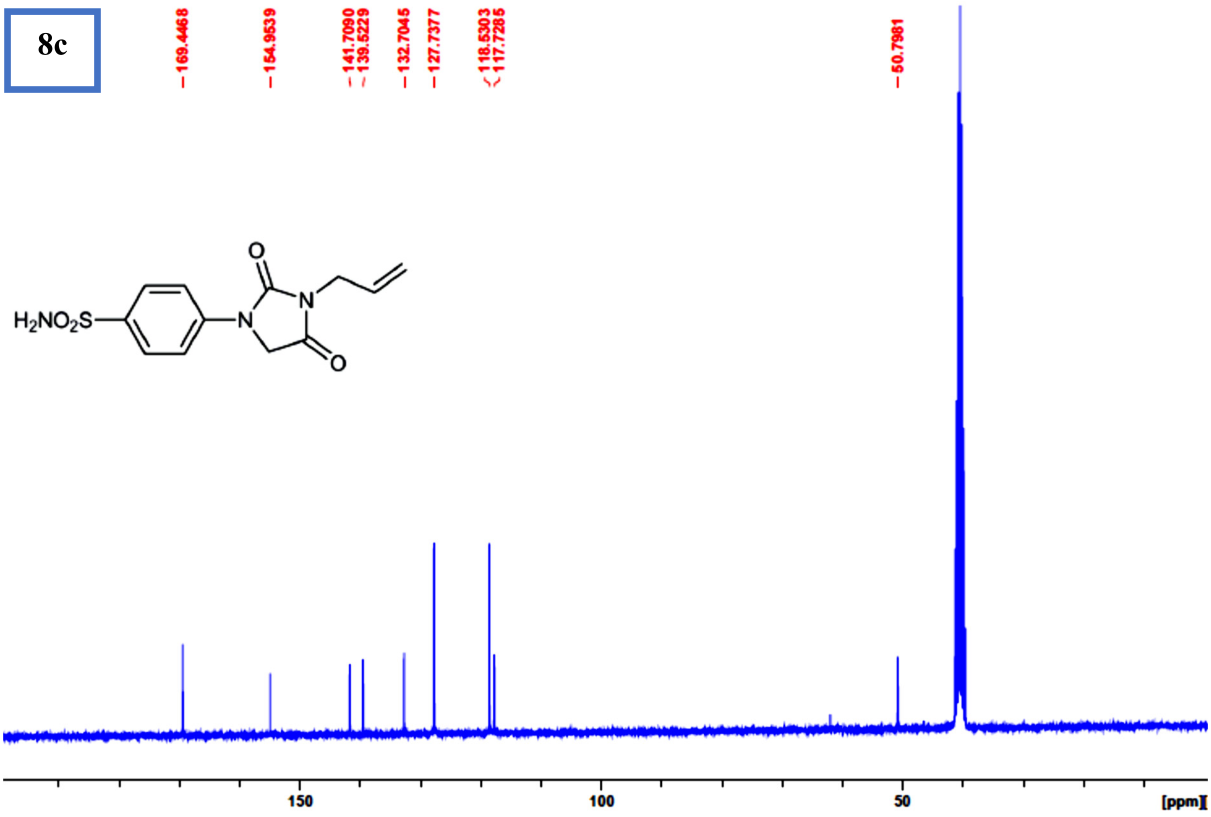
169.4468
154.9539
141.7090
139.5229
132.7045
127.7377
118.5303
117.7285
50.7981
H2NO2S
N
O
150
100
50
[ppm]

8d

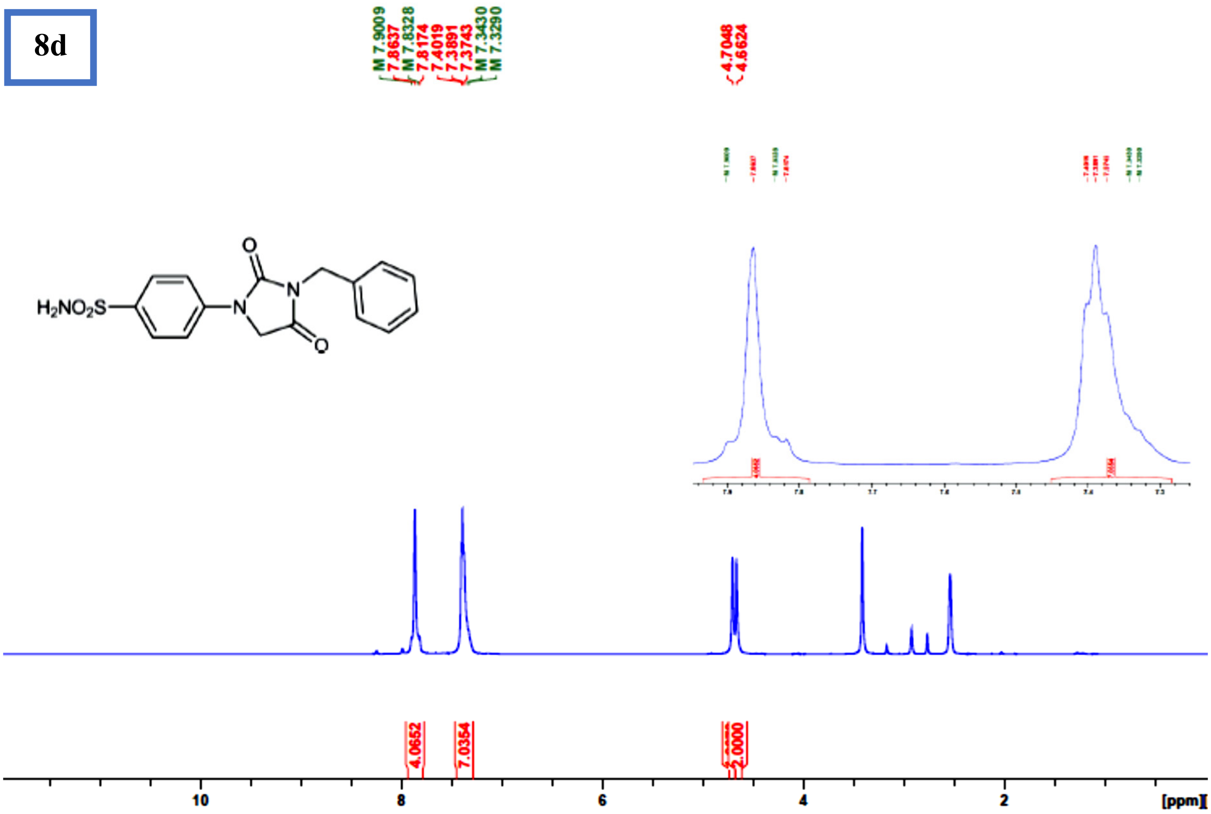
M 7.9009
7.8637
M 7.8328
7.8174
7.4019
7.3891
7.3743
M 7.3430
M 7.3290
4.7048
4.6624
H_2NO_2S
N
N
O
O
4.0652
7.0354
2.0000
10
8
6
4
2
[ppm]

8d

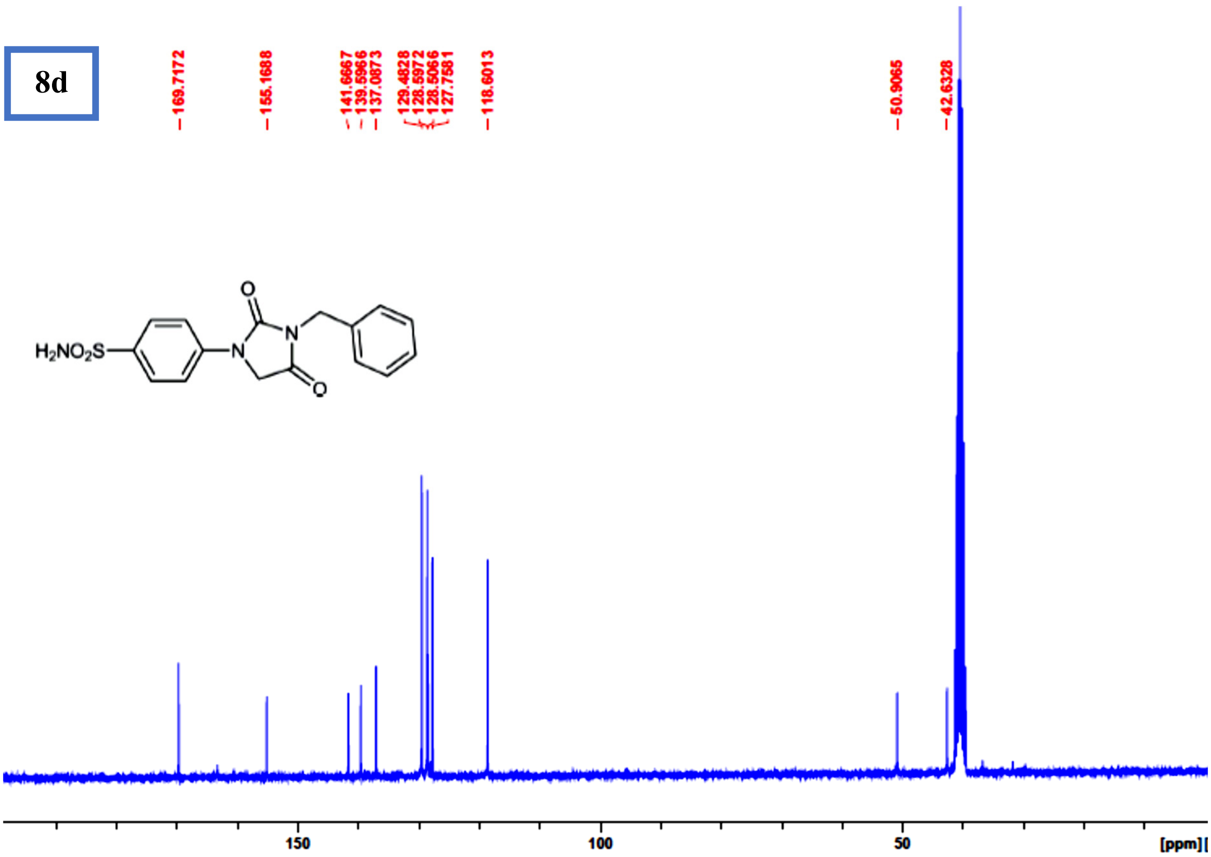
169.7172
155.1688
141.6667
139.5966
137.0873
129.4828
128.5972
128.5066
127.7581
118.6013
50.9065
42.6328
H_2NO_2S
N
N
O
O
150
100
50
[ppm]

8e

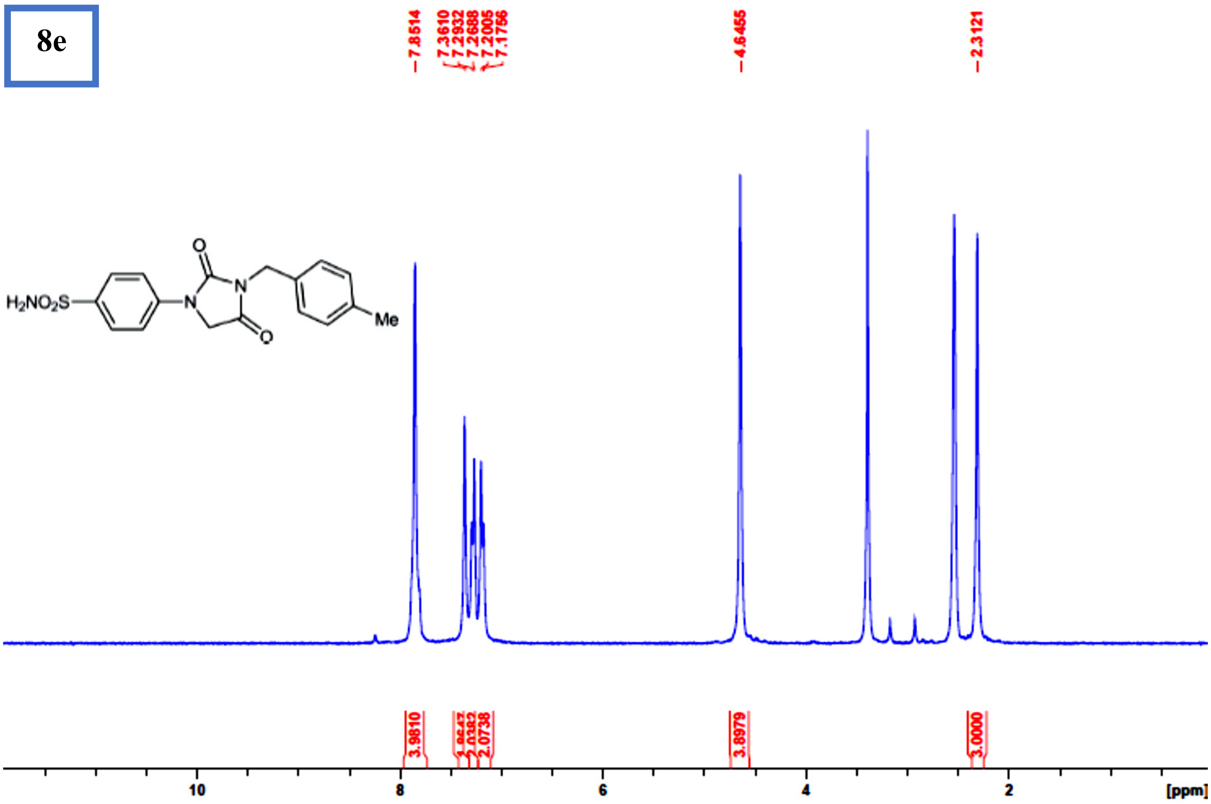

7.8514
7.3610
7.2932
7.2688
7.2005
7.1756
4.6455
2.3121
H2NO2S
N
N
O
O
Me
3.9810
2.0382
2.0738
3.8979
3.0000
10
8
6
4
2
[ppm]

8e

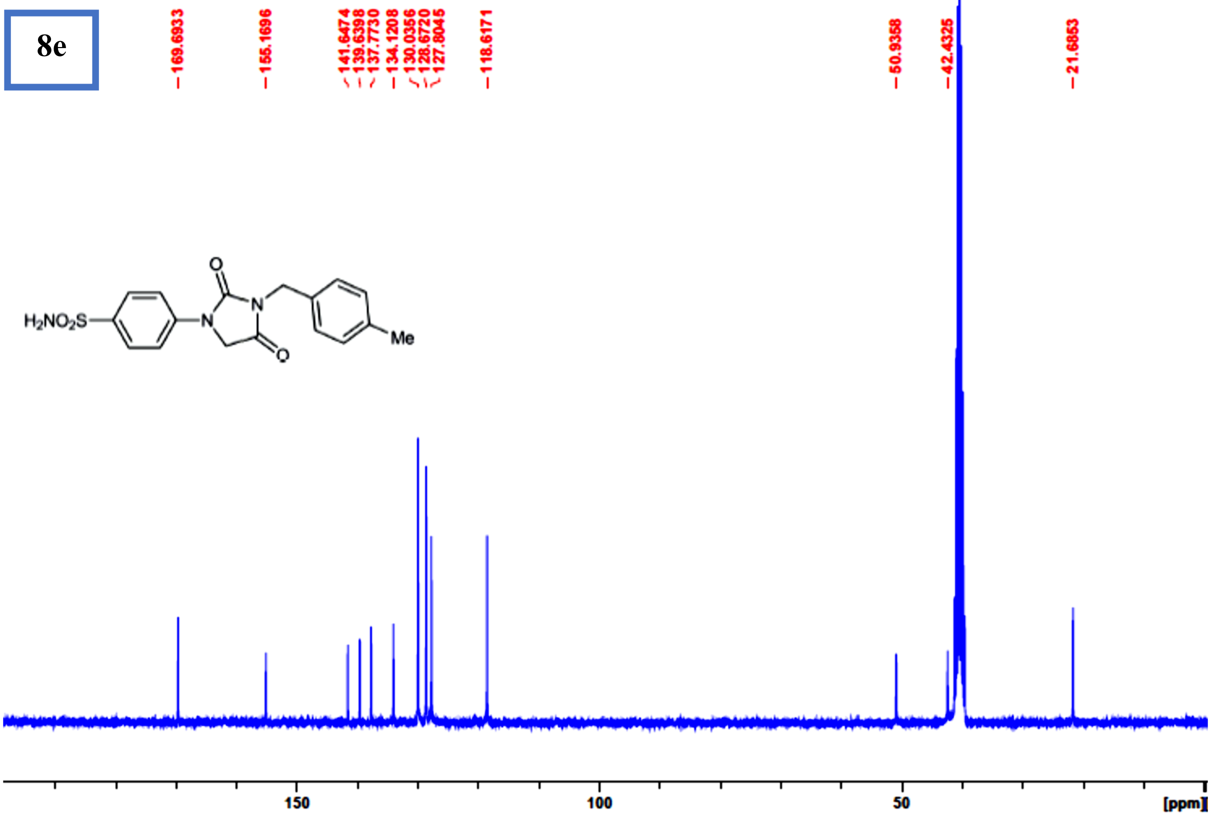

169.6933
155.1696
141.6474
139.6398
137.7730
134.1208
130.0356
128.6720
127.8045
118.6171
50.9358
42.4325
21.6853
H2NO2S
N
N
O
O
Me
150
100
50
[ppm]

8f

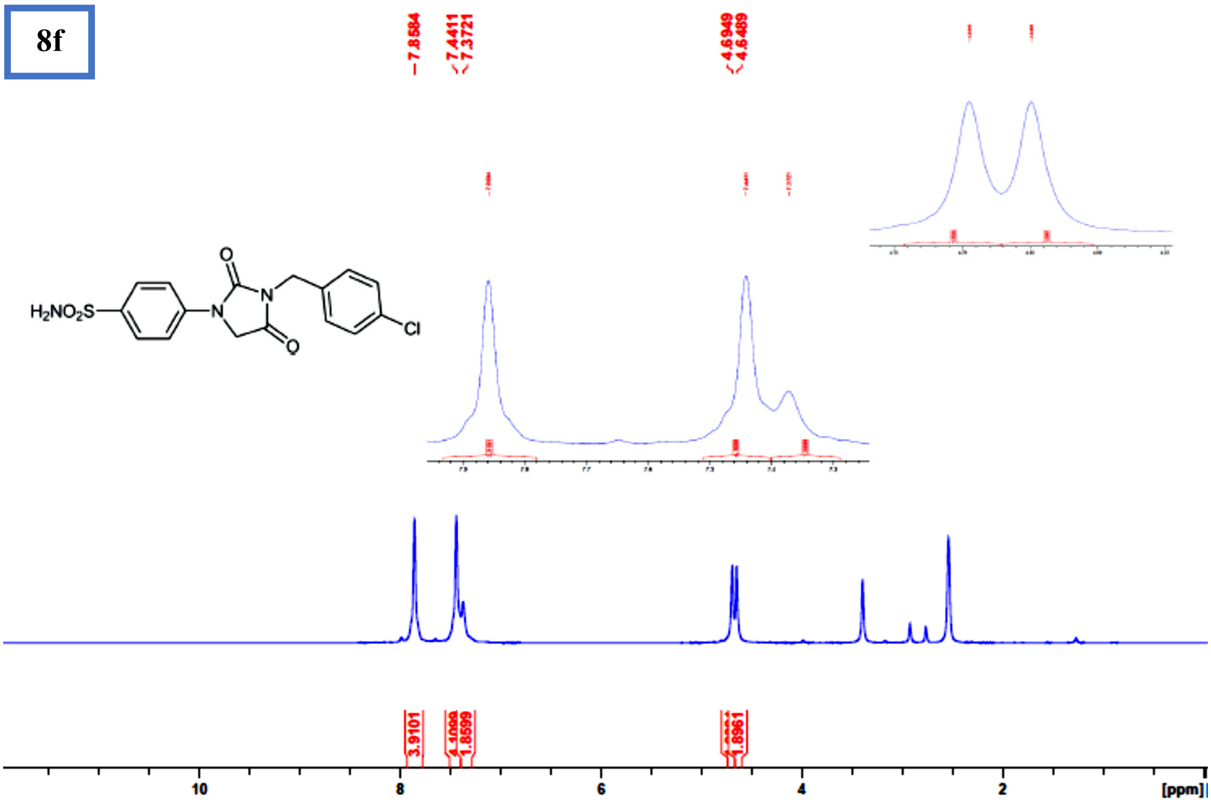
7.8584
7.4411
7.3721
4.6949
4.6489
H_2NO_2S
N
O
Cl
3.9101
4.1099
1.8599
1.8961
10
8
6
4
2
[ppm]

8f

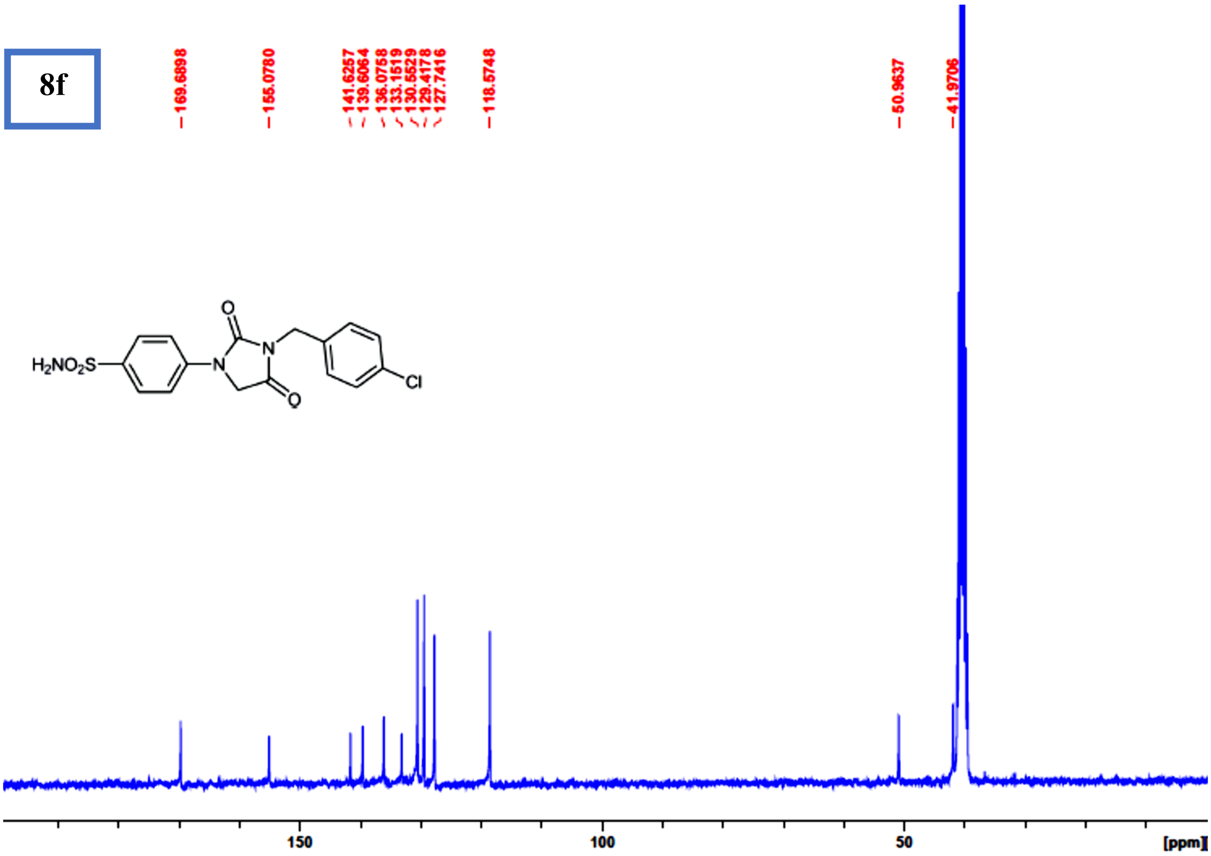
169.6898
155.0780
141.6257
139.6064
136.0758
133.1519
130.5529
129.4178
127.7416
118.5748
50.9637
41.9706
H_2NO_2S
N
O
Cl
150
100
50
[ppm]

8g

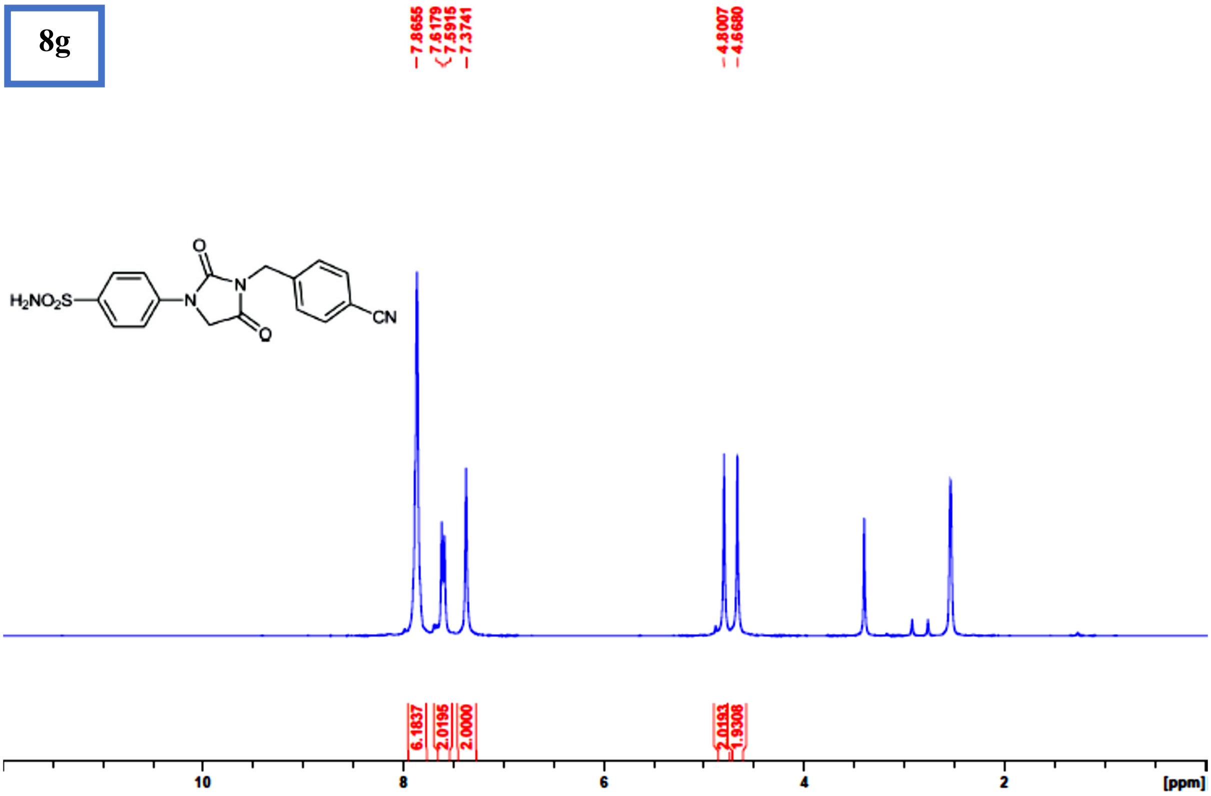
7.8655
7.6179
7.5915
7.3741
4.8007
4.6680
H2NO2S
N
N
O
O
CN
6.1837
2.0195
2.0000
2.0193
1.9308
10
8
6
4
2
[ppm]

8g

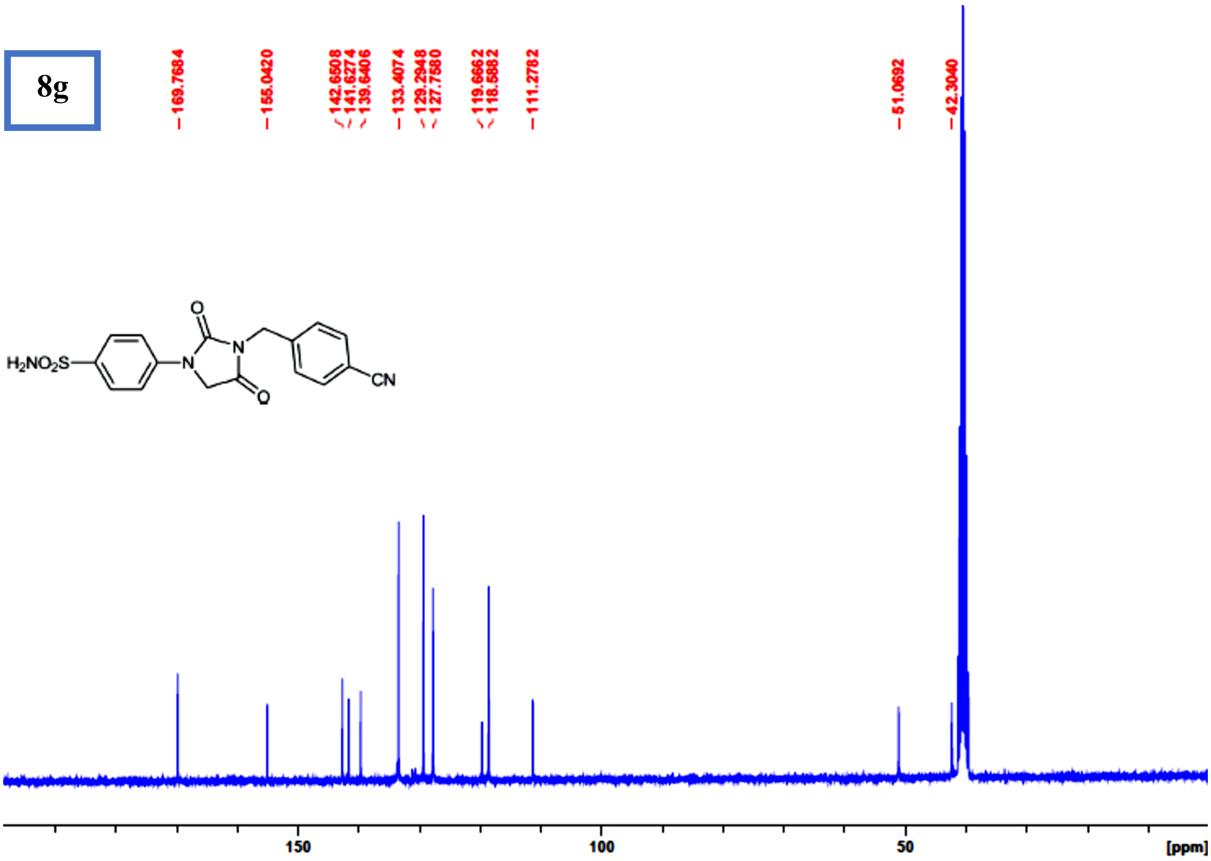
169.7684
155.0420
142.5508
141.6274
139.6406
133.4074
129.2948
127.7580
119.6662
118.5882
111.2782
51.0692
42.3040
H2NO2S
N
N
O
O
CN
150
100
50
[ppm]

8h

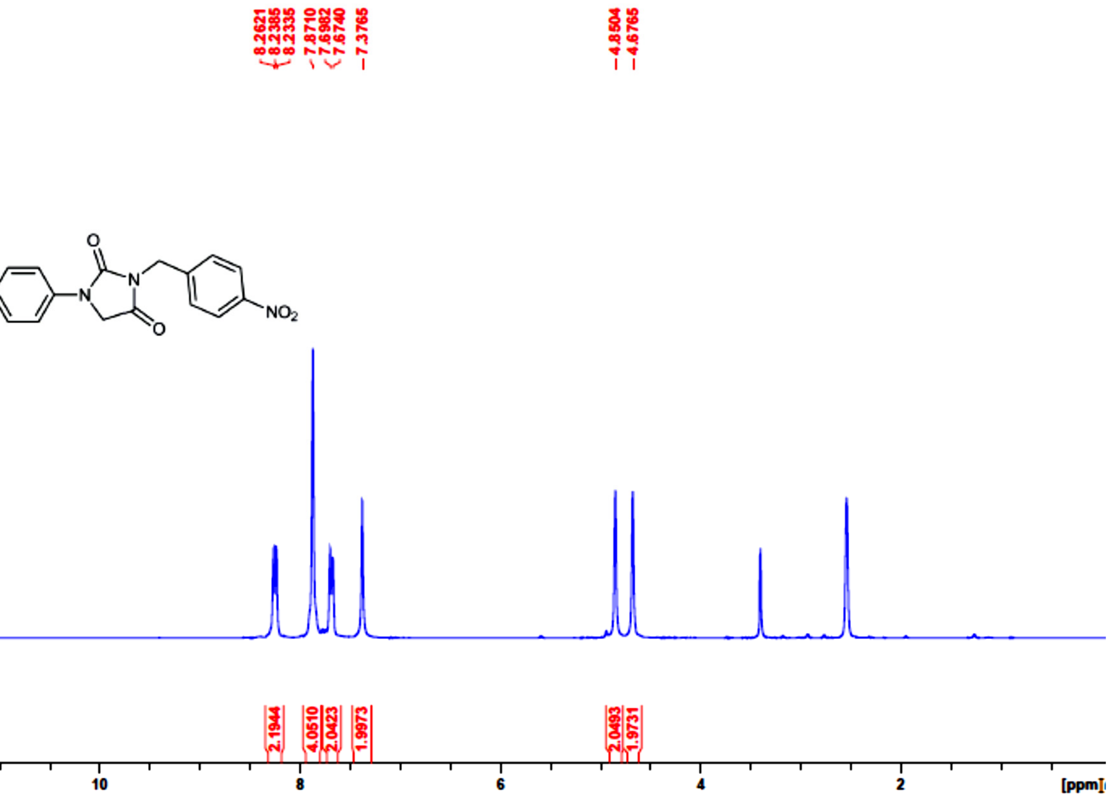
8.2621
8.2385
8.2335
7.8710
7.6982
7.6740
7.3765
4.8504
4.6765
H2NO2S
N
N
O
O
NO2
2.1944
4.0510
2.0423
1.9973
2.0493
1.9731
10
8
6
4
2
[ppm]

8h

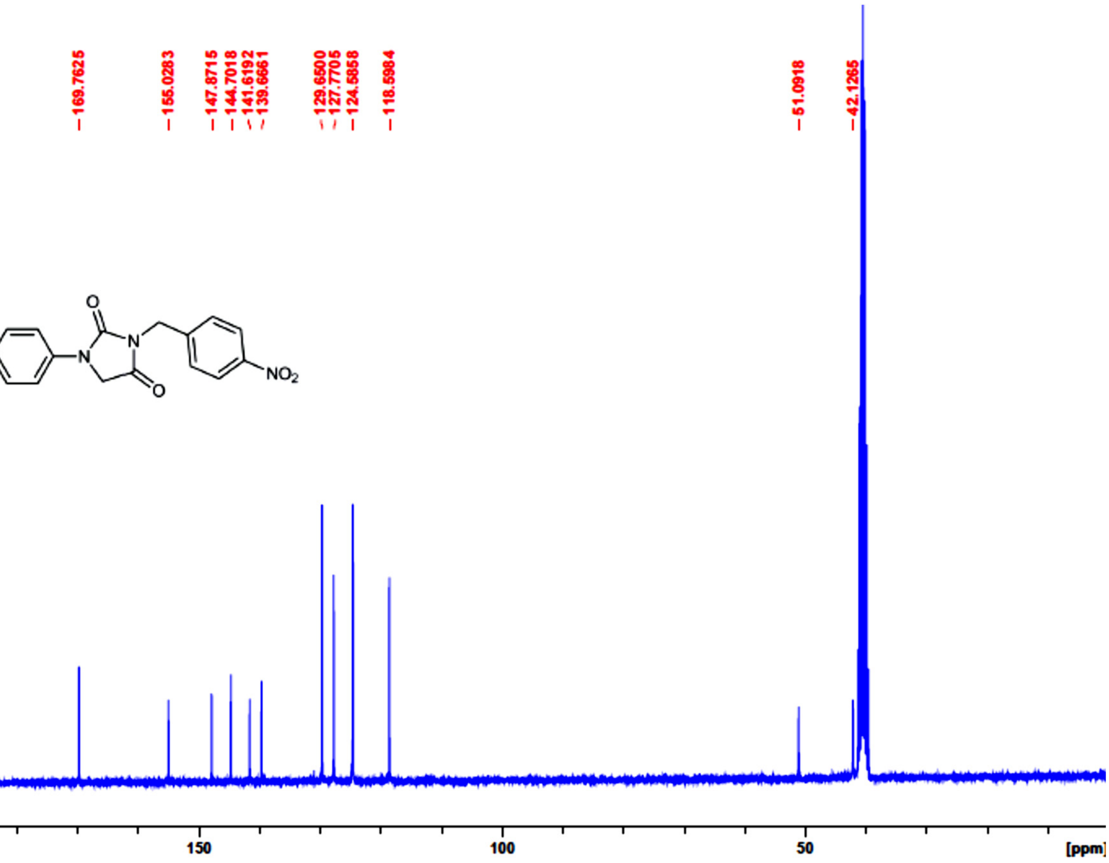
169.7625
155.0283
147.8715
144.7018
141.6192
139.6661
129.6500
127.7705
124.5858
118.5984
51.0918
42.1265
H2NO2S
N
N
O
O
NO2
150
100
50
[ppm]

8i

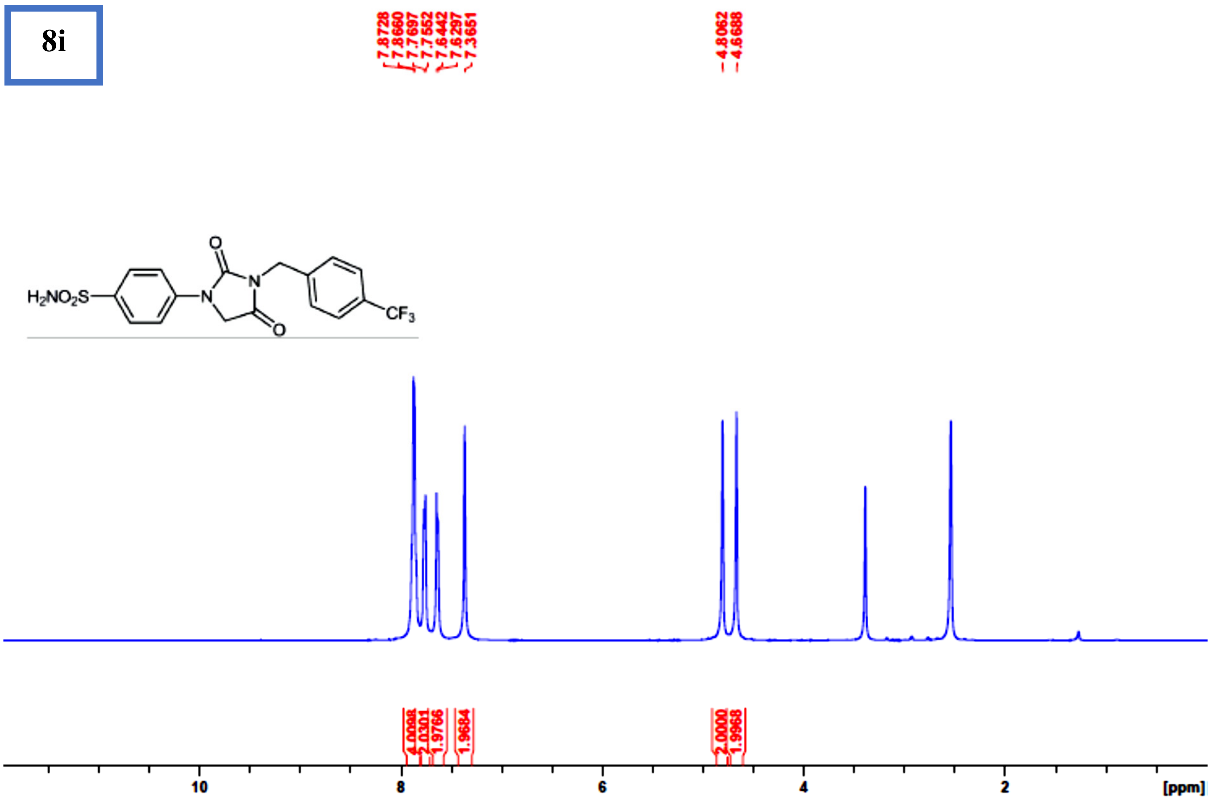

7.8728
7.8660
7.7697
7.7552
7.6442
7.6297
7.3651
4.8062
4.6688
H2NO2S
N
N
O
O
CF3
4.0098
2.0301
1.9766
1.9684
2.0000
1.9968
10
8
6
4
2
[ppm]

8i

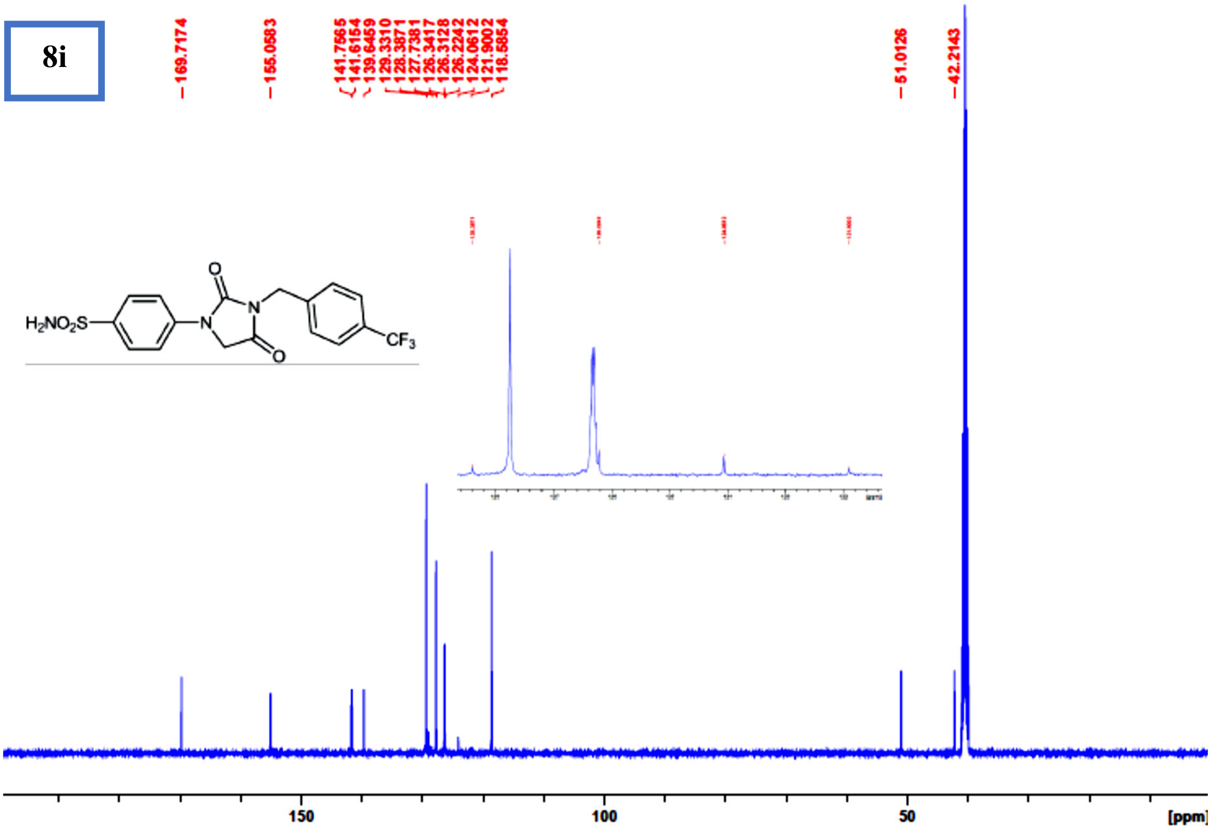

169.7174
155.0583
141.7565
141.6154
139.6459
129.3310
128.3871
127.7381
126.3417
126.3128
126.2242
124.0612
121.9002
118.5854
51.0126
42.2143
H2NO2S
N
N
O
O
CF3
150
100
50
[ppm]

8i

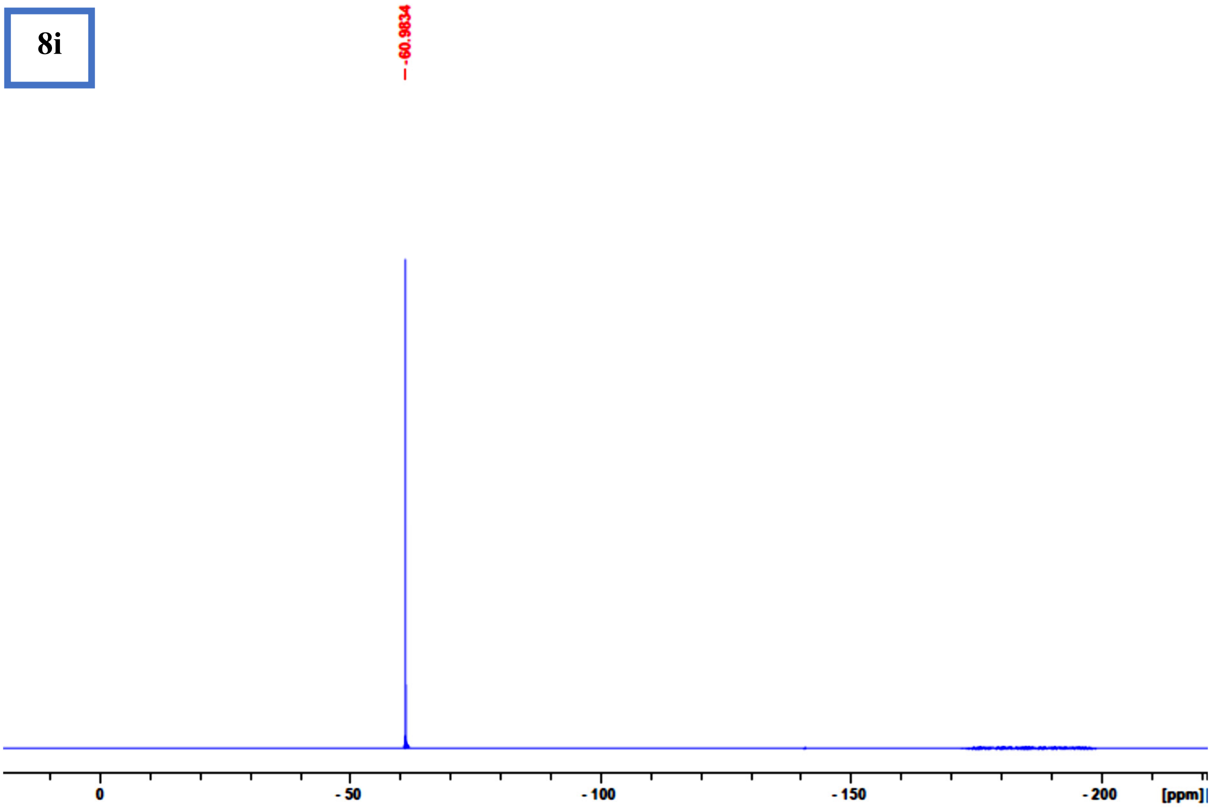
-60.9834
0
- 50
- 100
- 150
- 200
[ppm]

8j

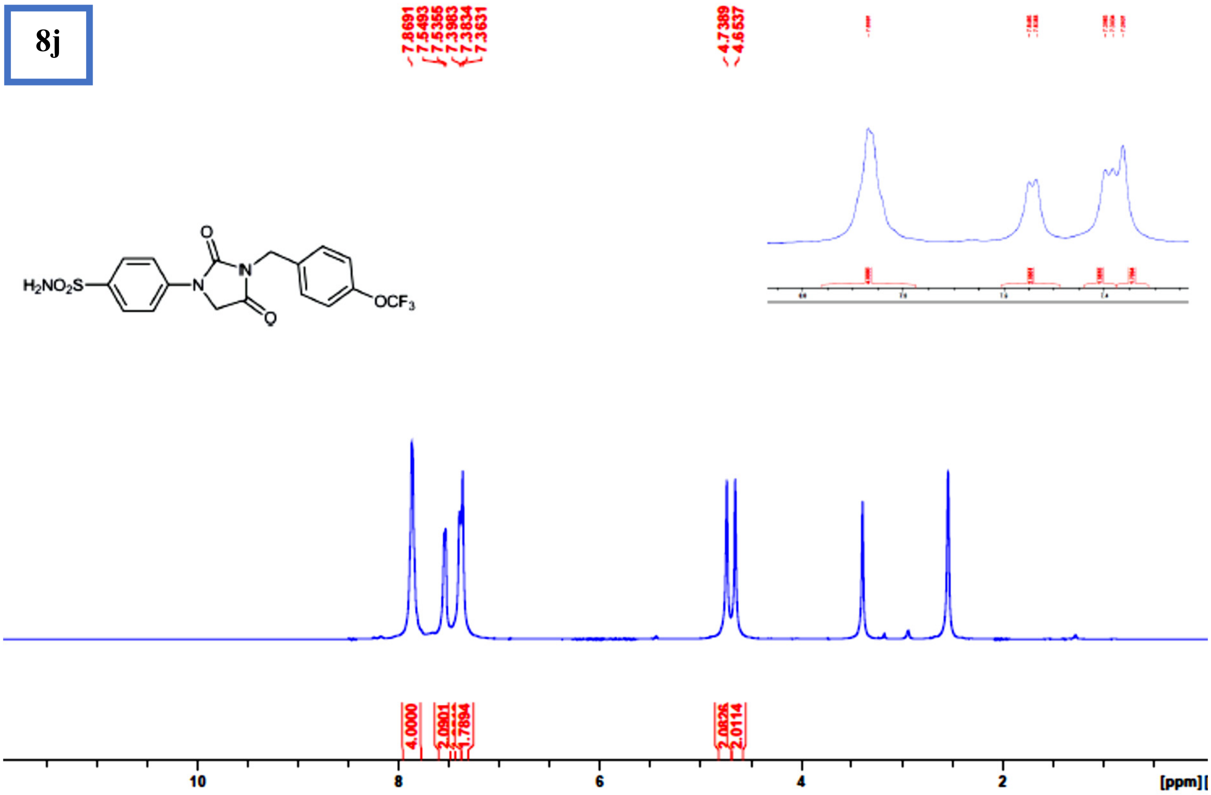
7.8691
7.5493
7.5355
7.3983
7.3834
7.3631
4.7389
4.6537
H_2NO_2S
O
N
N
O
OCF_3
4.0000
2.0901
1.7894
2.0826
2.0114
10
8
6
4
2
[ppm]

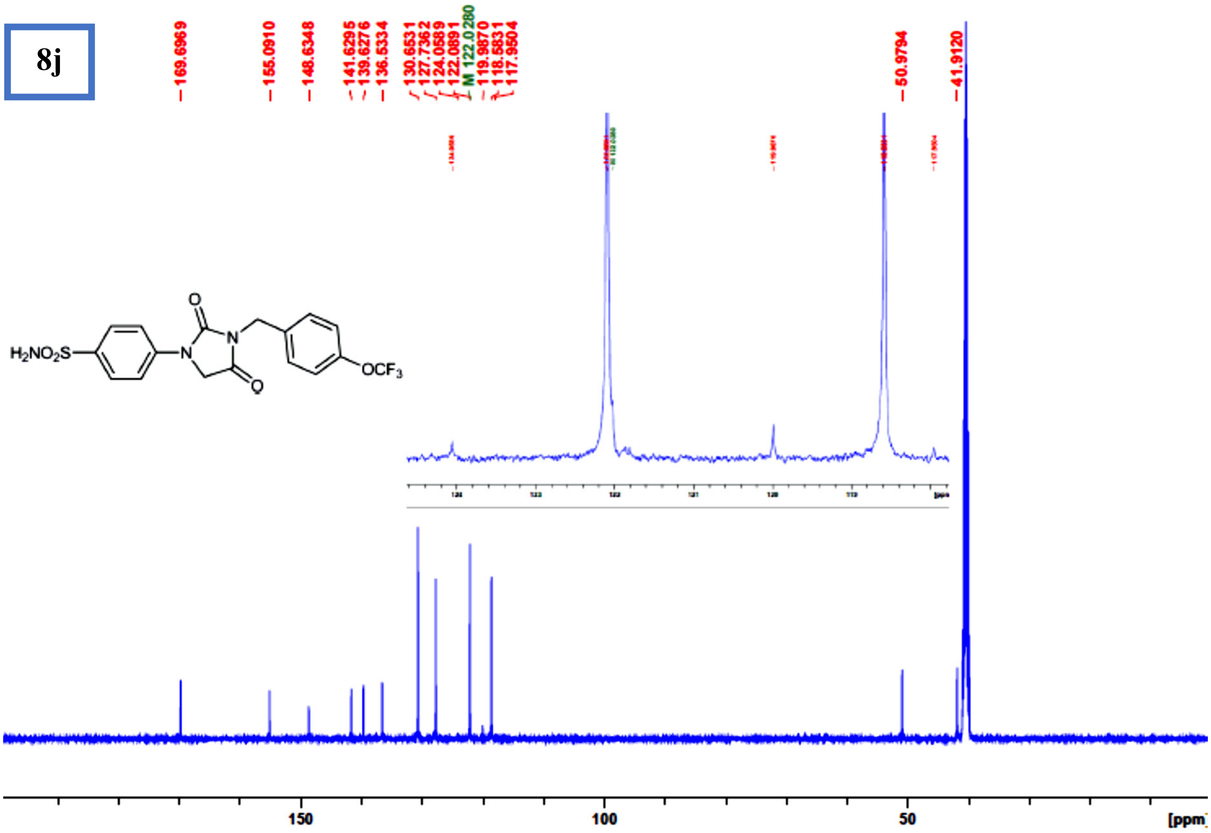
8j
169.6969
155.0910
148.6348
141.6295
139.6276
136.5334
130.6531
127.7362
124.0589
122.0891
M 122.0280
119.9870
118.5831
117.9504
50.9794
41.9120
H_2NO_2S
N
O
OCF$_3$
150
100
50
[ppm]

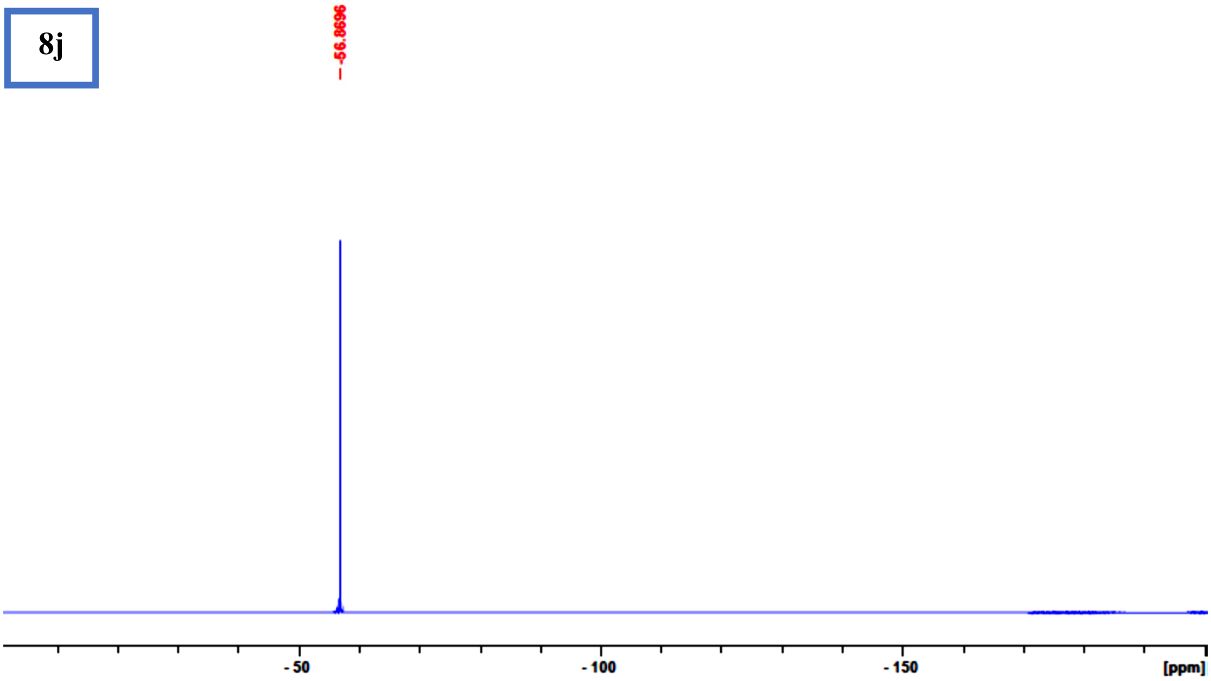
8j
-56.8696
- 50
- 100
- 150
[ppm]

8k

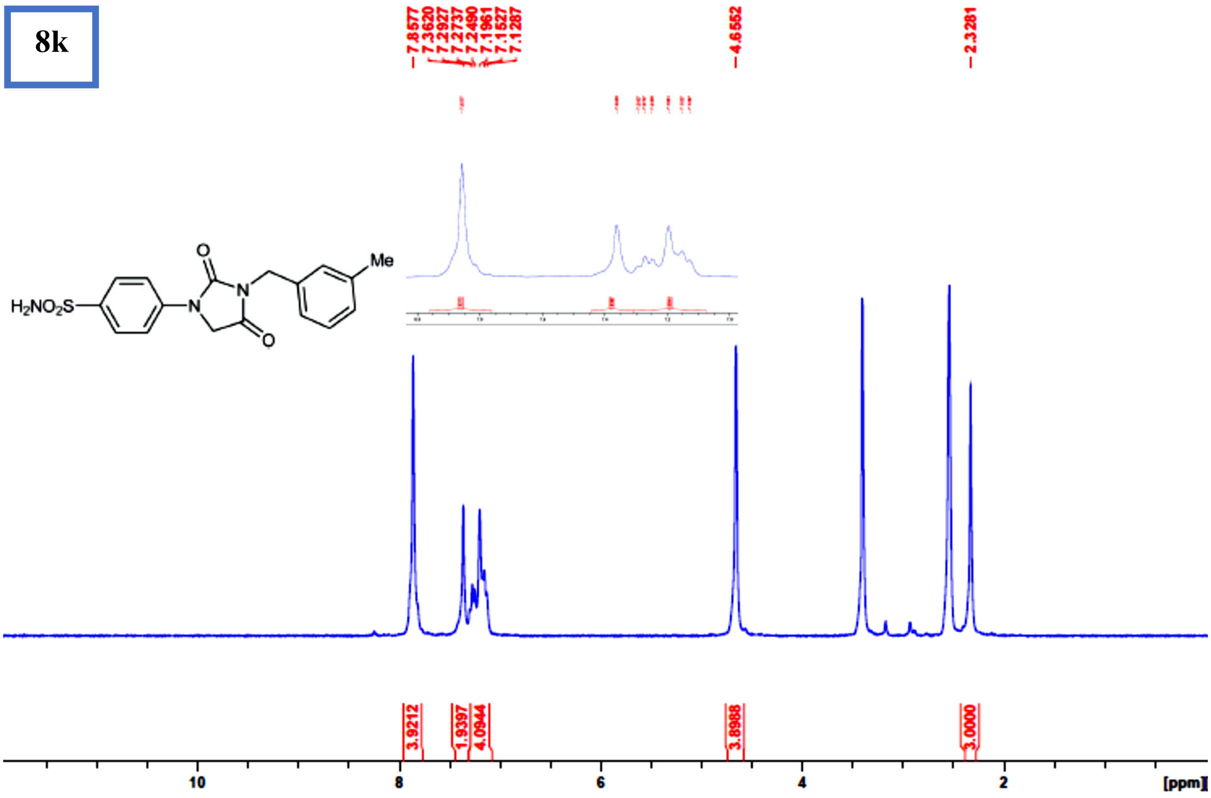

7.8577
7.3620
7.2927
7.2737
7.2490
7.1961
7.1527
7.1287
4.6552
2.3281
H2NO2S
N
N
O
O
Me
3.9212
1.9397
4.0944
3.8988
3.0000
10
8
6
4
2
[ppm]

8k

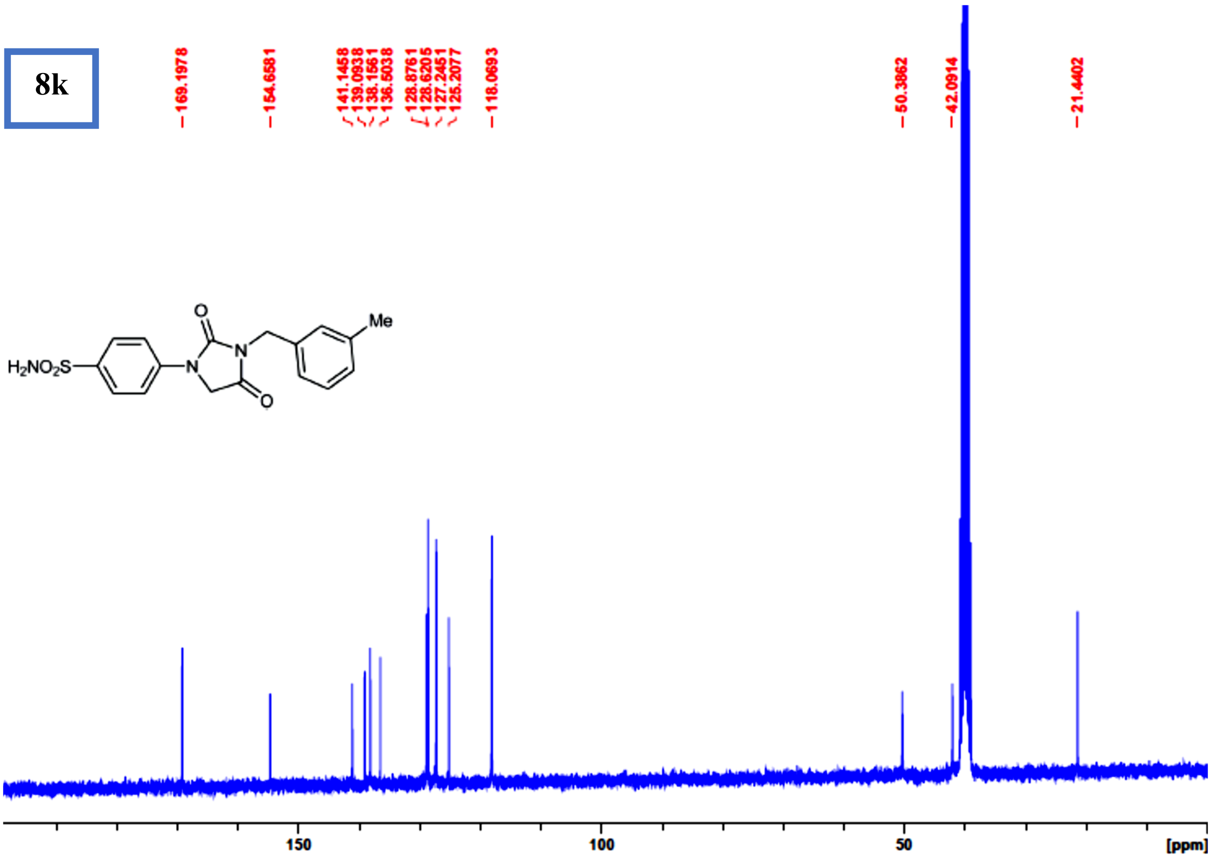

169.1978
154.6581
141.1458
139.0938
138.1561
136.5038
128.8761
128.6205
127.2451
125.2077
118.0693
50.3862
42.0914
21.4402
H2NO2S
N
N
O
O
Me
150
100
50
[ppm]

8l

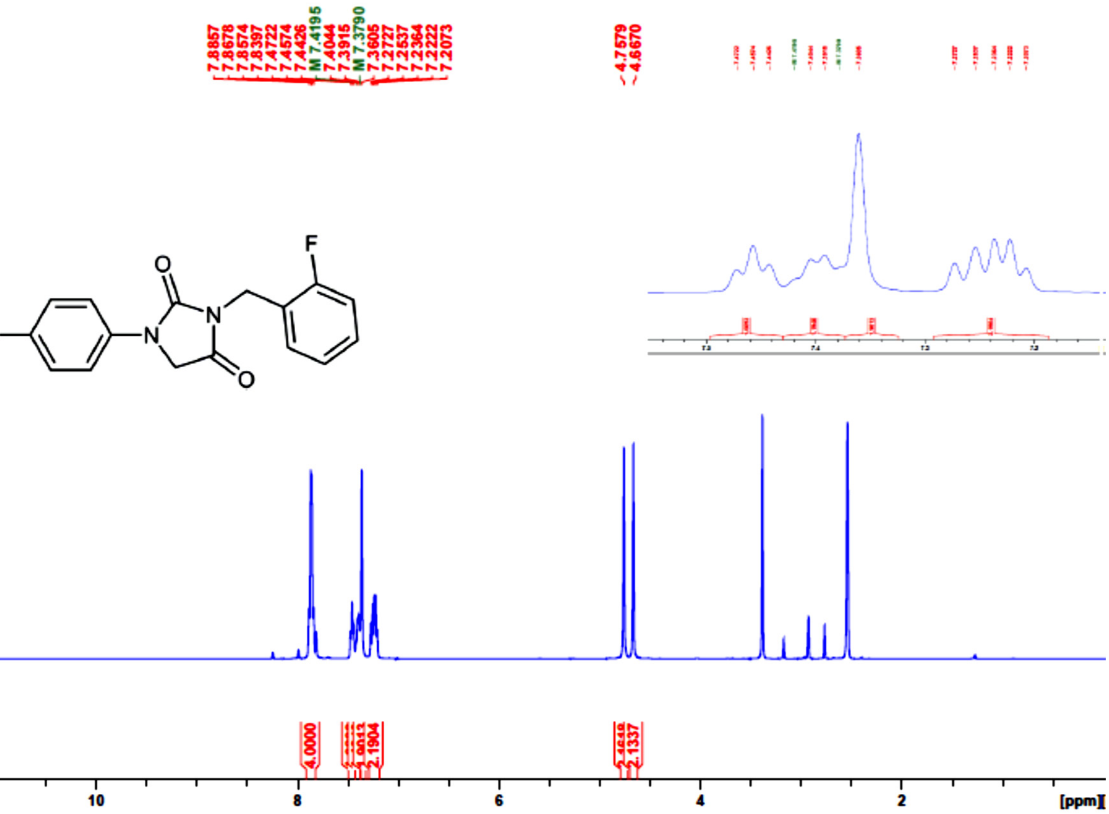
7.8857
7.8678
7.8574
7.8397
7.4722
7.4574
7.4426
M 7.4195
7.4044
7.3915
M 7.3790
7.3605
7.2727
7.2637
7.2364
7.2222
7.2073
4.7579
4.6670
F
O
N
H2NO2S
O
4.0000
2.1904
2.1337
10
8
6
4
2
[ppm]

8l

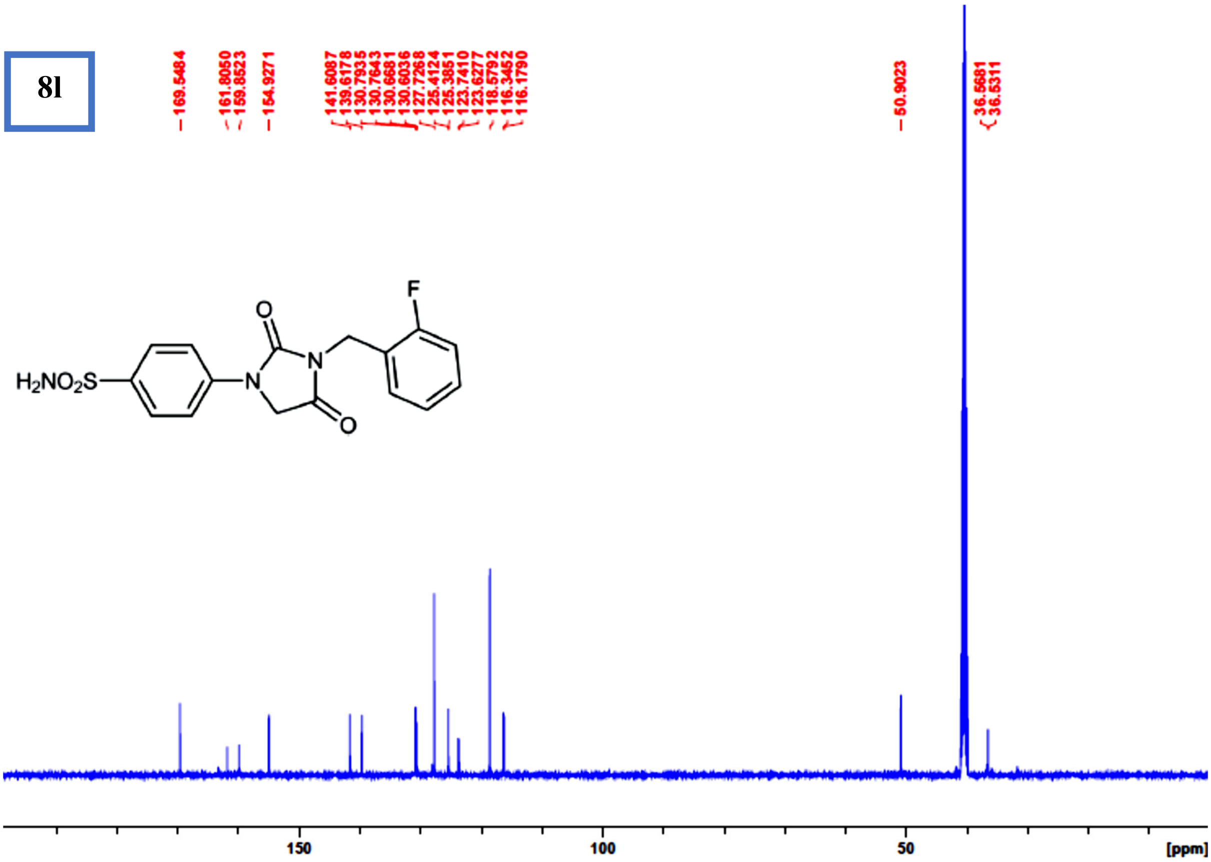
169.5484
161.8050
159.8523
154.9271
141.6087
139.6178
130.7935
130.7643
130.6681
130.6036
127.7268
125.4124
125.3851
123.7410
123.6277
118.5792
116.3452
116.1790
50.9023
36.5681
36.5311
F
O
N
H2NO2S
O
150
100
50
[ppm]

8l

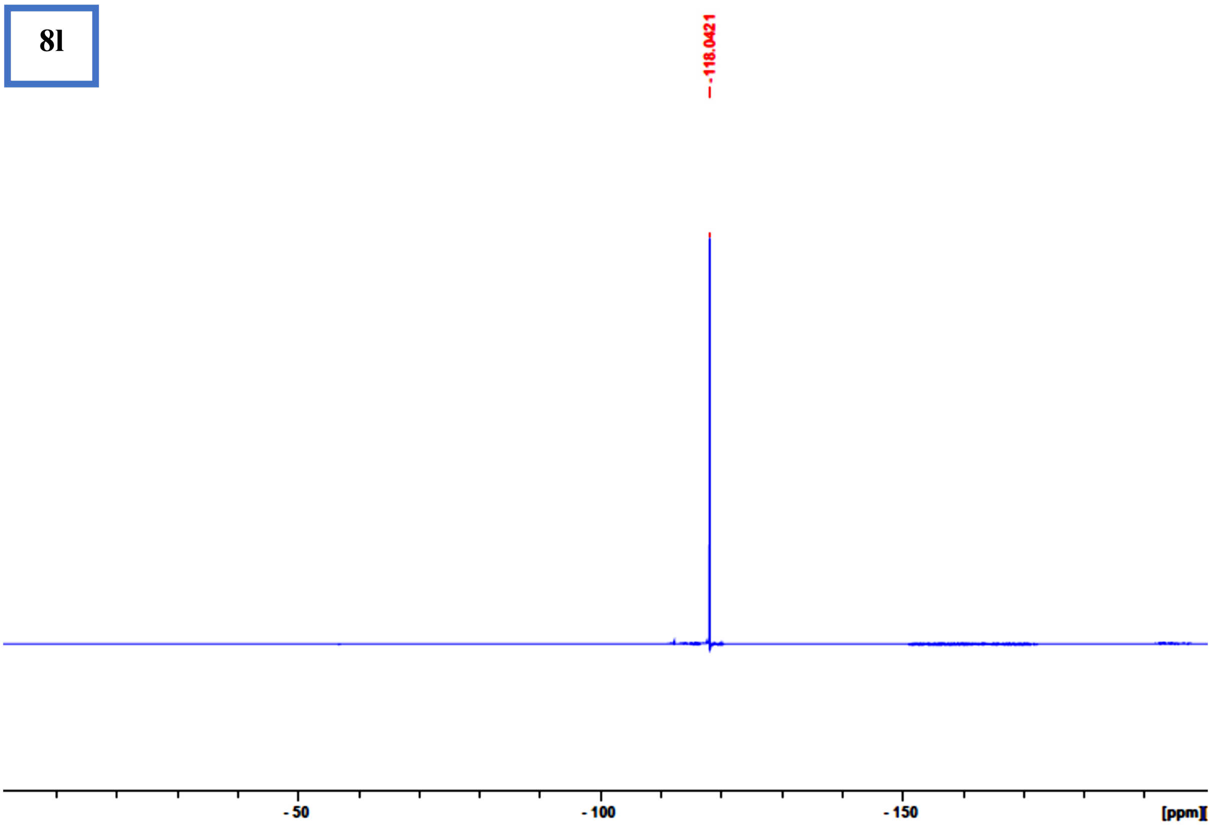
-118.0421
-50
-100
-150
[ppm]

8m

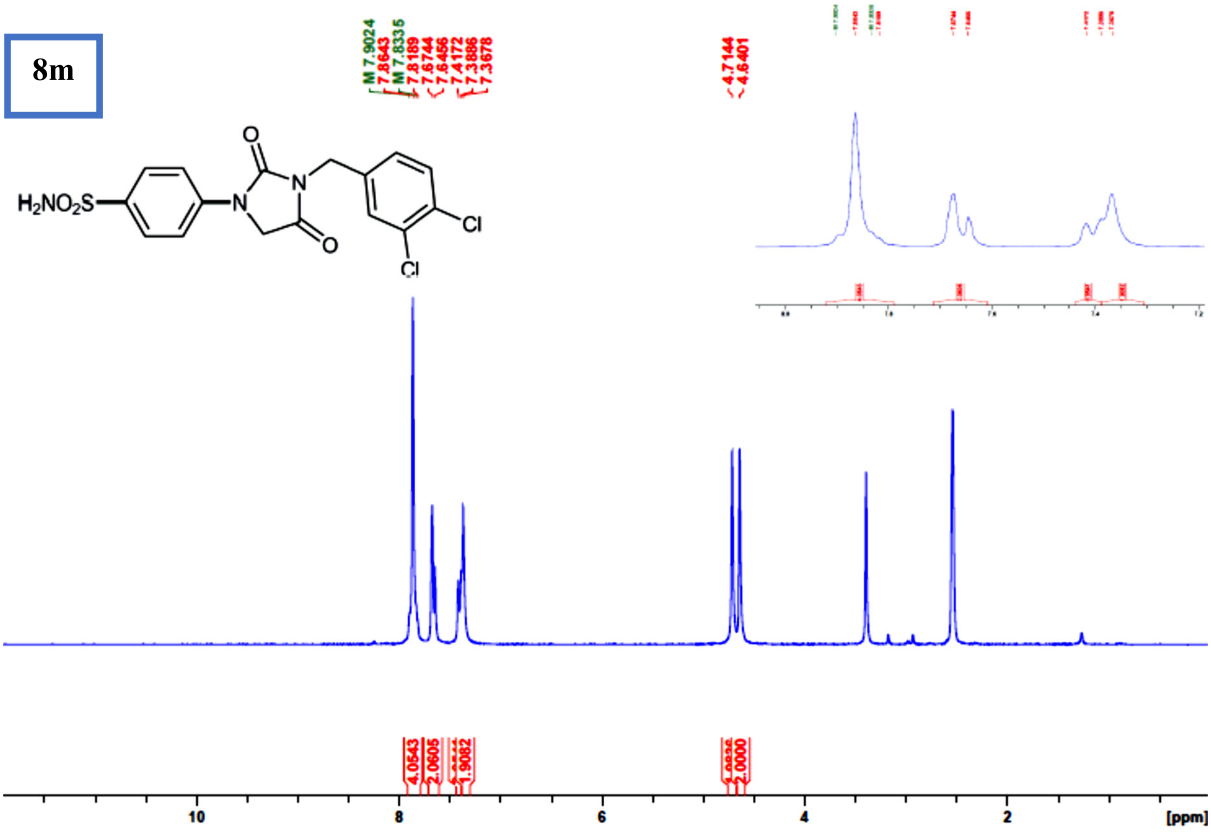
M 7.9024
7.8643
M 7.8335
7.8189
7.6744
7.6456
7.4172
7.3886
7.3678
4.7144
4.6401
H2NO2S
N
O
Cl
4.0543
2.0605
1.9082
2.0000
10
8
6
4
2
[ppm]

8m

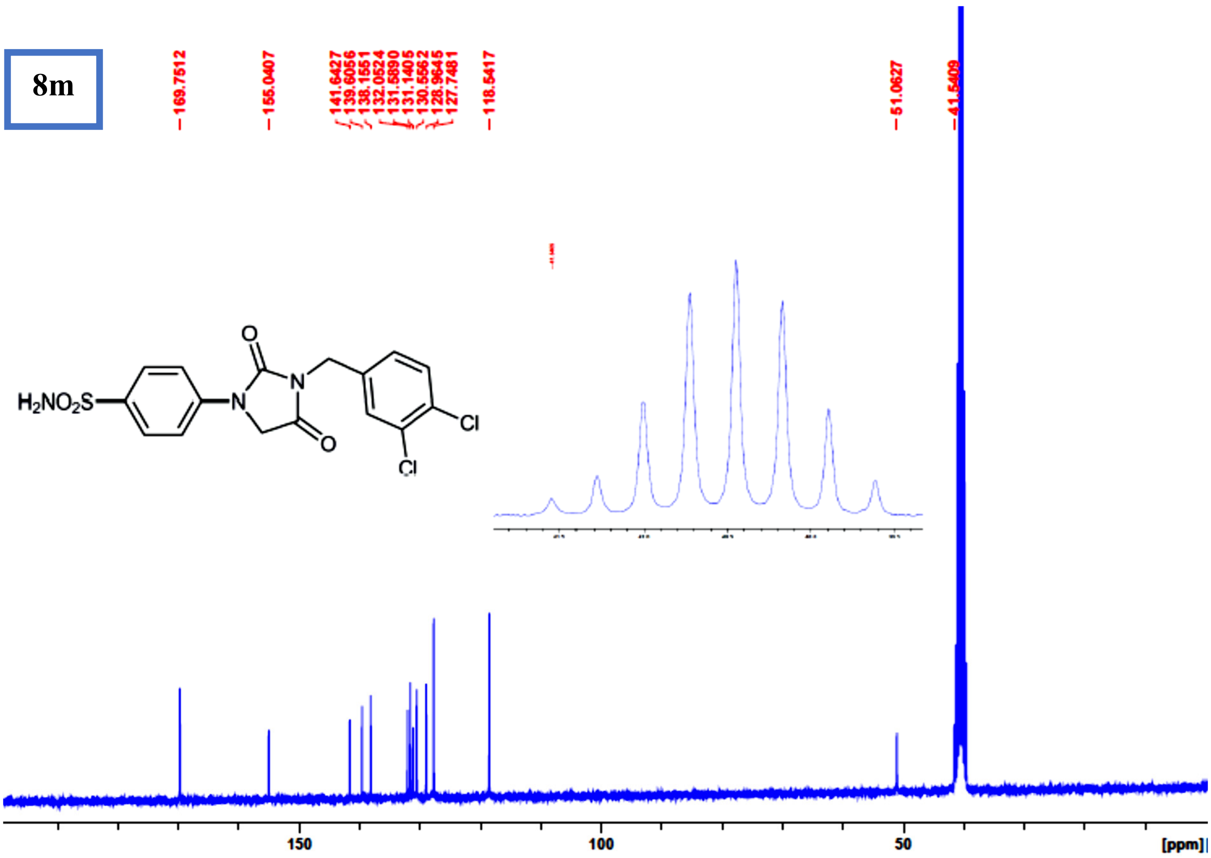
169.7512
155.0407
141.6427
139.6056
138.1551
132.0524
131.5890
131.1405
130.5562
128.9645
127.7481
118.5417
51.0627
41.5409
H2NO2S
N
N
O
O
Cl
Cl
150
100
50
[ppm]

8n

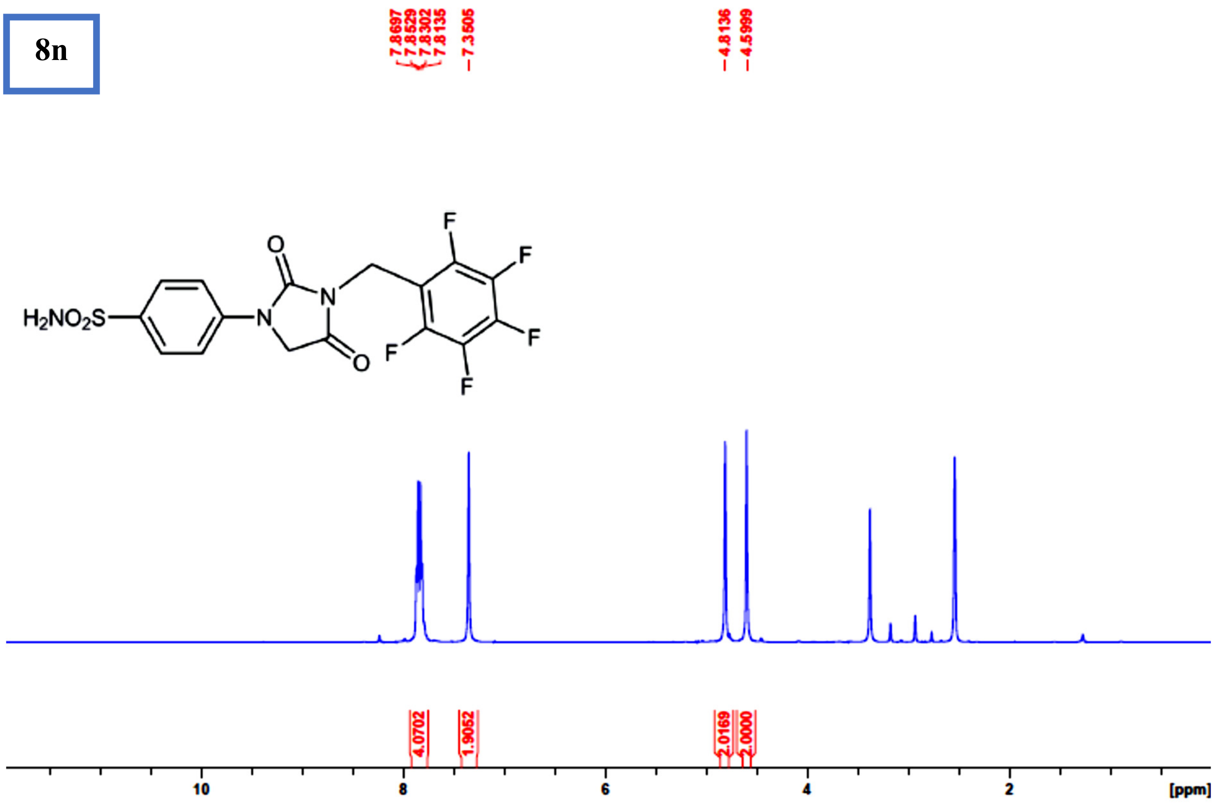
7.8697
7.8529
7.8302
7.8135
7.3505
4.8136
4.5999
H2NO2S
N
N
O
O
F
F
F
F
F
4.0702
1.9052
2.0169
2.0000
10
8
6
4
2
[ppm]

8n

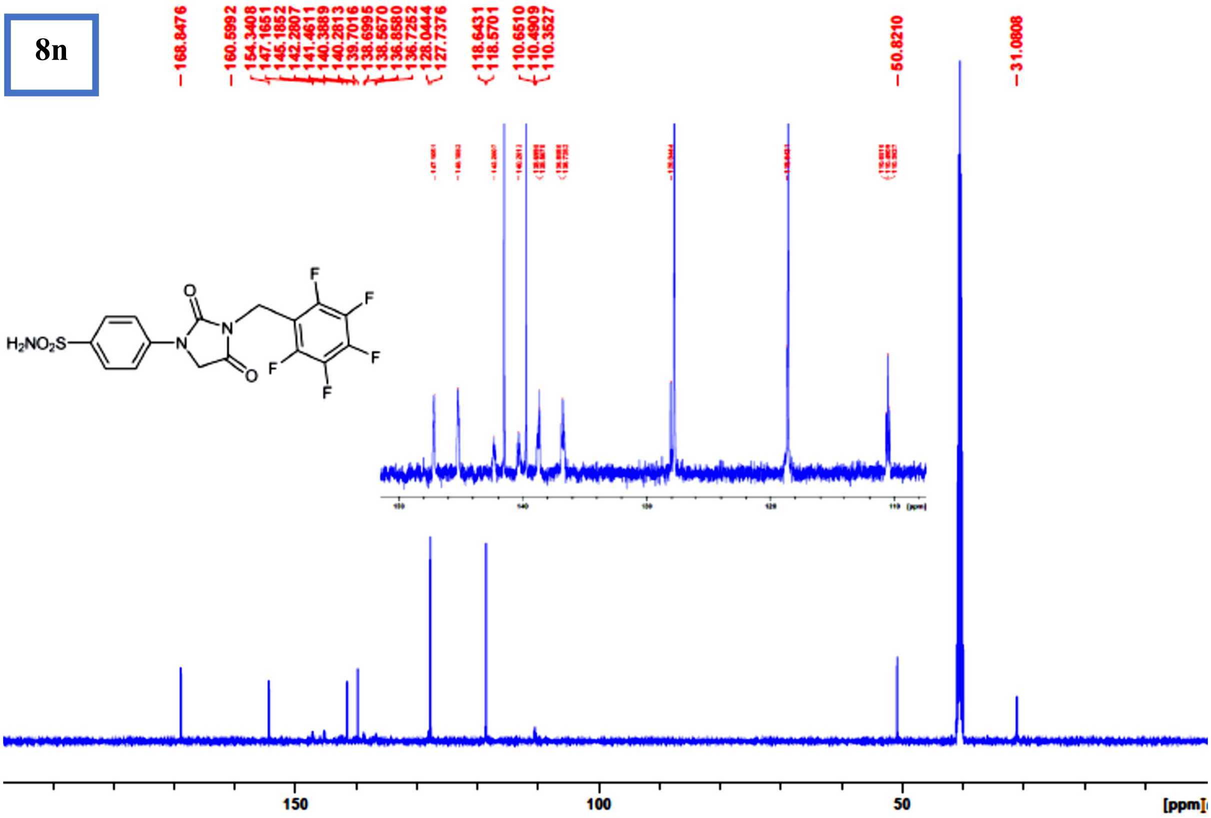
168.8476
160.5992
154.3408
147.1651
145.1852
142.2807
141.4611
140.3889
140.2813
139.7016
138.6995
138.5670
136.8580
136.7252
128.0444
127.7376
118.6431
118.5701
110.6510
110.4909
110.3527
50.8210
31.0808
H2NO2S
N
N
O
O
F
F
F
F
F
150
100
50
[ppm]

8n

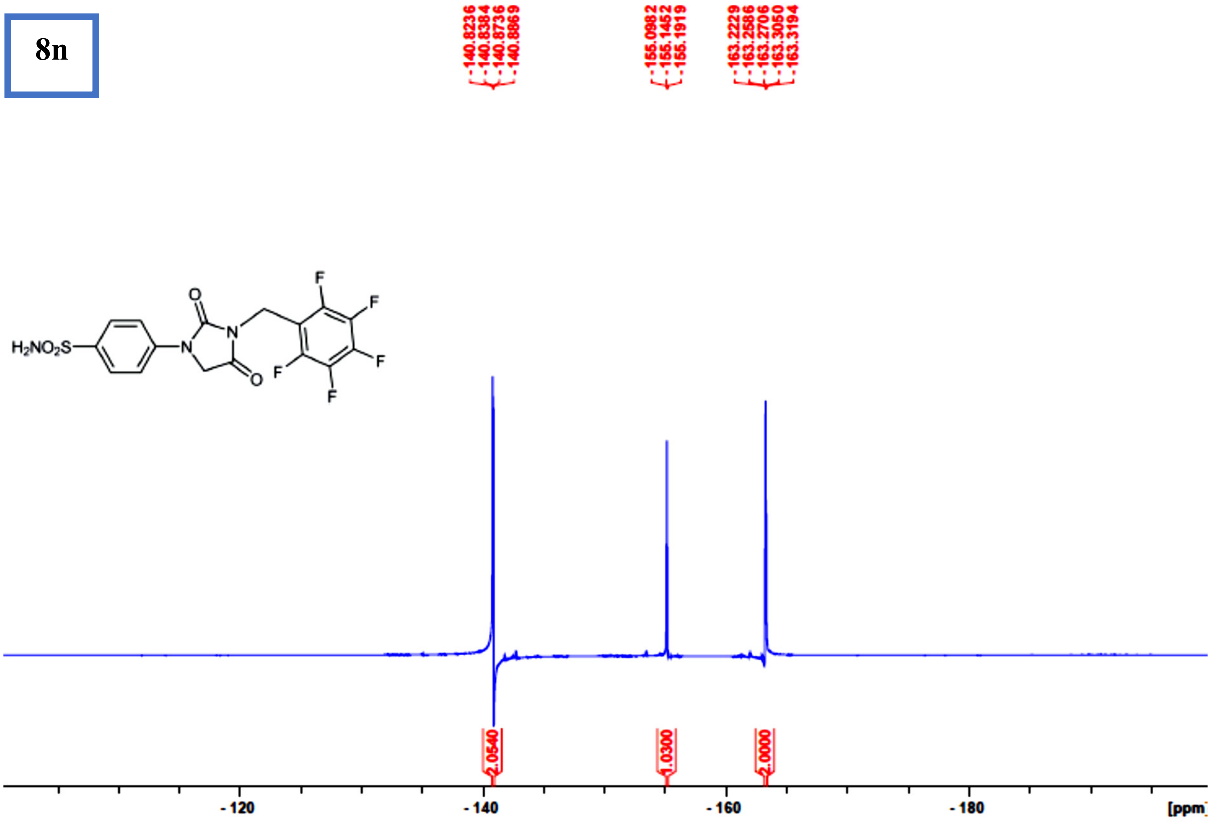
-140.8236
-140.8384
-140.8736
-140.8869
-155.0982
-155.1452
-155.1919
-163.2229
-163.2586
-163.2706
-163.3050
-163.3194
H2NO2S
N
N
O
O
F
F
F
F
F
2.0540
1.0300
2.0000
-120
-140
-160
-180
[ppm]
